# The Influence of Social Determinants of Health, Environmental, and Healthcare Resources on Life Expectancy in the Organization of Islamic Cooperation (OIC) Countries: A Systematic Review

**DOI:** 10.3390/ijerph23040531

**Published:** 2026-04-18

**Authors:** Ruhina Aimaq, Hana AlSumri, Amal S. Malehi, Zainab M. Al-Zadjali, Kouthar S. Al-Alawi, Laila S. Al-Saadi, Rawan Ibrahim, Sumaiya Al Aamri, Rabab Mohammed Bedawi Husien, Anak Agung Bagus Wirayuda, Moon Fai Chan

**Affiliations:** 1 Department of Family Medicine & Public Health, College of Medicine & Health Sciences, Sultan Qaboos University, Muscat 123, Oman; s155924@student.squ.edu.om (R.A.); alsumry@squ.edu.om (H.A.); or zainab.alzadjali@yahoo.com (Z.M.A.-Z.); kautheral-alawi@hotmail.com (K.S.A.-A.); s156586@student.squ.edu.om (R.I.); s156810@student.squ.edu.om (S.A.A.); s136159@student.squ.edu.om (R.M.B.H.); 2 Ministry of Education, Muscat 100, Oman; laila50767@gmail.com; 3 Department of Medicine, Faculty of Medicine and Health, Institut Teknologi Sepuluh Nopember, Surabaya 60111, Indonesia; anak.wirayuda@its.ac.id

**Keywords:** systematic review, life expectancy, socioeconomic factors, health resources, environmental factors, social determinants of health, OIC countries

## Abstract

**Highlights:**

**Public health relevance—How does this work relate to a public health issue?**

- Life expectancy (LE) is a fundamental indicator for assessing the performance and effectiveness of public health systems.
- Persistent gaps in LE between and within countries now mirror broader social and environmental inequalities rather than purely biomedical risks.

**Public health significance—Why is this work of significance to public health?**

- Cross-country studies have examined determinants of LE globally; however, in some Organization of Islamic Cooperation (OIC) member states, existing evidence remains fragmented.
- No systematic review exists that collates and critically appraises quantitative evidence on how social determinants of health, environmental conditions, and healthcare resources jointly influence LE across OIC member countries.

**Public health implications—What are the key implications or messages for practitioners, policy makers, and/or researchers in public health?**

- Higher gross domestic product per capita, better education, stronger employment, and greater health expenditure are consistently associated with longer LE, whereas poverty, inequality, air pollution, and limited health resources tend to shorten lives or slow progress.
- These findings indicate that improving LE in OIC countries will require coordinated multisectoral policies rather than isolated interventions, and that future research should prioritize stronger causal designs and improved country- and subnational-level data to clarify mechanisms and support more targeted interventions.

**Abstract:**

Life expectancy (LE) varies widely across Organization of Islamic Cooperation (OIC) countries, reflecting differences in economic, social, environmental, and health-system conditions. This review aimed to synthesize quantitative evidence on determinants of LE at birth in OIC member countries. The study was conducted in accordance with the PRISMA guidelines, and a systematic search of electronic databases was performed up to September 2025. After screening 5312 records and assessing full texts, studies were appraised using the Joanna Briggs Institute checklists, with an inclusion threshold of ≥80%. A total of 54 studies, mainly ecological, time-series, and panel analyses using national-level data, were included. Higher gross domestic product per capita, education, employment, and health expenditure were consistently associated with longer LE. In contrast, poverty, income inequality, air pollution, and carbon dioxide emissions were associated with shorter LE. Clear differences were observed across World Bank income groups, with LE being lowest in low-income OIC countries and highest in high-income Gulf Cooperation Council states, where gains were driven more by health-system resources than by income growth. Improving LE in OIC countries requires integrated economic, social, environmental, and health-system policies.

## 1. Introduction

Life expectancy (LE) is the average number of years an individual is expected to live, assuming prevailing age-specific mortality rates remain unchanged, and it typically varies across geographical settings and time periods [1]. LE is a fundamental indicator for assessing the performance and effectiveness of public health systems [2]. The global LE appears to have declined by 0.92 years between 2019 and 2020 and by another 0.72 years between 2020 and 2021, but the decline seems to have ended during the last quarter of 2021 [3]. A recent study found that LE has increased steadily, reflecting progress in income, education, and healthcare; however, gains have been uneven across regions and have slowed or stalled in some settings [4]. Persistent gaps in LE, both between and within countries, now mirror broader social and environmental inequalities rather than purely biomedical risks [5].

The Organization of Islamic Cooperation (OIC) brings together 57 member states spanning four continents, with marked heterogeneity in economic development, demographic structures, and health-system capacity [6]. Recent data from the Statistical, Economic and Social Research and Training Centre for Islamic Countries (SESRIC) show that average LE in OIC countries increased from 62.2 years in 2000 to 68.9 years in 2023, yet remains below the global average of around 73.3 years, with wide dispersion across income groups and regions [7]. At the same time, population aging is accelerating: the share of adults aged 65+ in OIC countries rose from 3.6% in 1990 to 4.8% in 2021 and is projected to reach 9.5% by 2050, placing additional demands on health and social protection systems [8]. Policy analyses further indicate that OIC countries, particularly low- and lower-middle-income members, tend to have lower LE at birth (LEB), higher maternal and under-five mortality, and more limited financial and physical access to essential health services than comparable non-OIC countries [9].

The World Health Organization (WHO) defines the social determinants of health (SDH) as the conditions in which people are born, grow, work, live, and age, shaped by the distribution of power, money, and resources [10]. A growing body of empirical work shows that LE is strongly influenced by social and economic conditions, including income, employment, education, social protection, and the broader welfare state, alongside individual health behaviors [5]. Cross-national analyses have identified economic prosperity, income inequality, labor market conditions, and social policy spending as important predictors of LE and mortality [4]. Within the OIC context, national-level modeling in BahrSain has demonstrated that macroeconomic factors, health status and resources, and sociodemographic structure jointly influence LE over time [11]. Alongside social and economic determinants, environmental conditions, especially ambient and household air pollution, have emerged as major drivers of premature mortality and reduced LE. Global estimates suggest that exposure to fine particulate matter (PM2.5) and other pollutants shortens average LE by roughly 1–3 years, with particularly large impacts in low- and middle-income countries [12,13].

Health-system resources and service coverage constitute a third critical domain influencing LE. Comparative analyses indicate that higher and more efficient health expenditures, universal health coverage, adequate health workforce density, and access to quality primary care are generally associated with longer LE, although the strength and shape of these relationships vary by context [4,14]. In Middle East and North Africa (MENA) countries, including several Gulf Cooperation Council GCC and broader OIC members, increased per capita health spending has been positively correlated with gains in LE at birth, but with notable inefficiencies and cross-country differences [14]. Policy reports on OIC member states highlight that many countries invest a lower share of gross domestic product (GDP) in health, rely heavily on out-of-pocket payments, and face shortages of nurses and midwives compared with non-OIC peers, especially in low-income settings [6,9].

Although numerous cross-country studies have examined determinants of LE globally, in some OIC member states, existing evidence remains fragmented. Most analyses focus on a single domain (for example, health spending or environmental degradation) or a single country or subregion, use different sets of covariates, and apply heterogeneous methods, making it difficult for policymakers in OIC countries to draw coherent conclusions. Recent longitudinal and reviews have begun to organize determinants of LE into broad categories such as healthcare expenditures, social spending, health behaviors, and environmental risks, but these syntheses have not been tailored to the specific socioeconomic, demographic, and health-system realities of OIC members [4,15,16]. To date, no systematic review has collated and critically appraised quantitative evidence on how social determinants of health, environmental conditions, and healthcare resources jointly influence LE across OIC member countries.

To address this gap, the present systematic review will synthesize observational and longitudinal studies reporting associations between LE and social, environmental, and health-system determinants in OIC member states, with attention to regional and income-group heterogeneity and methodological quality. By integrating evidence across these three domains, the review aims to identify quantitative studies examining the association between LE and social, environmental, and healthcare resource determinants in OIC countries, clarify which modifiable factors can be targeted through policy, and highlight priority gaps for future empirical research.

## 2. Materials and Methods

### 2.1. Search Strategies

This systematic review protocol was registered with PROSPERO (CRD420251164403), and the methods align with PRISMA (Preferred Reporting Items for Systematic Reviews and Meta-Analyses) 2020 [17] (Supplementary File S2). A comprehensive search of international bibliographic databases was undertaken, including MEDLINE (via PubMed), Scopus, Web of Science, and ScienceDirect, as well as multidisciplinary and subject-specific databases (e.g., Academic Search Complete, Business Source Complete, IEEE Xplore) and Google Scholar for additional material. In addition, selected regional databases (such as Al Manhal) were searched to improve coverage of studies from OIC countries. The EBSCOhost platform served as the primary search engine available through the university library.

All databases were searched from their respective dates up to 3 September 2025, with no restrictions imposed on the earliest year of publication. Only peer-reviewed full-text articles published in English were eligible. No restrictions were placed on publication status, except that the report must contain original quantitative data. Because the review focuses specifically on OIC member states, the search strategy was applied separately to each of the 57 OIC countries. The search strategy combined terms related to LE with terms describing socioeconomic, macroeconomic, environmental, and health-system determinants. Both free-text keywords and database-specific subject headings (e.g., MeSH terms in MEDLINE) were used where applicable, and search strings were adapted to the syntax of each database. The core Boolean structure combined determinants using “OR”, which were then linked to the outcome and country terms using “AND”. The general search structure was as follows.

The same core Boolean operator “AND” and “OR” was used for all countries: “life expectancy” AND (“socioeconomic status” OR “sociodemographics” OR “macroeconomics” OR “health resources” OR “environmental” OR “pollution” AND [“country name”]). A sample of our full search strategy is provided in Supplementary File S1.

### 2.2. Inclusion and Exclusion Criteria

Studies were included if conducted in at least one OIC member country at the national, subnational, or city level. They involved the general population or a clearly defined population-based sample. To be eligible, articles had to quantitatively assess at least one determinant within the domains of social and sociodemographic factors (such as income, poverty, inequality, education, unemployment, labor market indicators, fertility, urbanization, or household characteristics), macroeconomic and structural factors (such as GDP per capita or social and health expenditures), environmental factors (including air pollution indices like PM_2.5_, environmental quality scores, carbon emissions, or climate-related indicators), or healthcare resources and health-system factors (including total or public health expenditure, health workforce density, hospital beds per 1000 population, or coverage of essential services). The primary outcome of interest was LE, preferably LE at birth, whether total or sex-specific; studies reporting LE at specific ages or closely related summary measures were also considered if they reported LE at birth or if the determinant outcome relationships could reasonably be compared. Only quantitative observational or ecological designs (cross-sectional, cohort, longitudinal, time-series, or panel analyses using primary or secondary data) that provided extractable quantitative effect estimates, or sufficient data to derive them, were included. These study designs are commonly used to examine population-level determinants of LE, as they allow analysis of macro-level indicators such as socioeconomic conditions, environmental exposures, and health-system resources across countries or over time [18,19].

Studies were excluded if they were case reports, case series, reviews, editorials, commentaries, conference abstracts, or methodological papers without original data. Qualitative papers or those that did not present extractable quantitative associations between determinants and LE were also excluded. For multi-country analyses combining OIC and non-OIC settings, inclusion was restricted to studies that presented results for OIC countries separately or allowed extraction from tables, figures, or Supplementary Materials.

### 2.3. Study Selection

**Figure 1:**
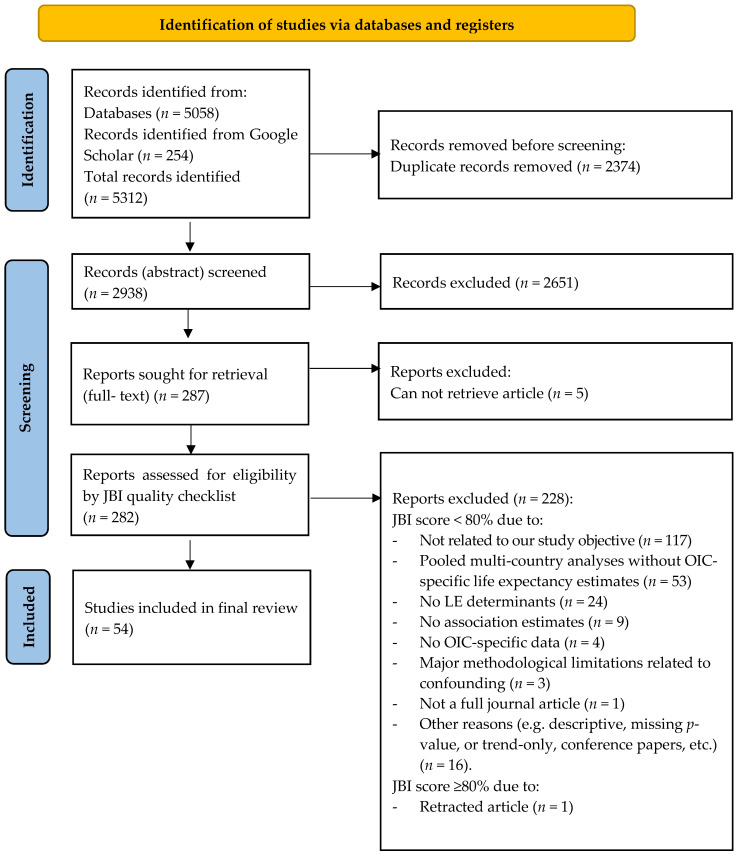
PRISMA flow diagram.

All references retrieved from the database searches were first exported to Zotero [20], where duplicate records were identified and removed. Study selection was carried out in two stages. In the first stage, the titles and abstracts of all records were screened against the predefined inclusion and exclusion criteria by the first author (RA). Studies that clearly did not relate to LE, did not involve any OIC member country, or did not examine relevant social, environmental, or healthcare determinants were excluded at this point. As part of the pilot screening, inter-rater reliability was assessed using a two-way consistency intraclass correlation coefficient (ICC). Agreement across six reviewers for 13 studies was good (single-measure ICC = 0.86, 95% CI 0.73–0.95), supporting the consistency of the study selection process. In the second stage, the full texts of all potentially eligible articles were retrieved and independently reviewed by the authors (RA, ZZ, KA, LS, RI, SA, RMBH, and AABW) using the same eligibility criteria; reasons for exclusion at the full-text stage (such as absence of a LE outcome, non-OIC setting, inadequate quantitative data, or failure to report any association between LE and the determinants of interest) were documented in Figure 1. Any disagreements between reviewers at either stage were resolved through discussion and, where necessary, consultation within the review team. The overall process of identification, screening, eligibility assessment, and final inclusion of studies is presented in a PRISMA flow diagram, which shows the number of records at each stage and the main reasons for exclusion during full-text review.

### 2.4. Evaluation of the Quality of Reports on the Studies

Methodological quality was assessed using the appropriate Joanna Briggs Institute (JBI) Critical Appraisal Tool for each study design [21]. Each study was independently appraised by six reviewers (RA, ZZ, KA, RI, SA, and RMBH). For every item in the relevant JBI checklist, responses were recorded as “yes”, “no”, “unclear”, or “not applicable”. For each study, the number of “yes” responses was converted to a percentage, and a predefined inclusion threshold of 80% “yes” responses was applied; only studies meeting this criterion were retained for the final synthesis. In total, 54 studies achieved a JBI score of ≥80% and were included in the review to minimize the risk of bias and strengthen confidence in the identified determinants. This threshold is consistent with approaches adopted in other JBI-based evidence syntheses, which are classified as high methodological quality [22,23].

### 2.5. Data Extraction

Data from the 54 included studies were extracted in a standardized manner. For each study, basic characteristics (first author, year of publication, study design, country, study period, population, and sample size) were recorded, together with the main determinant domains, specific determinants, and their reported associations with LE. Determinants were classified a priori into four categories (social/sociodemographic, macroeconomic, environmental, and healthcare resources), and all available quantitative effect estimates (crude or adjusted coefficients, correlations, or other measures of association) with their corresponding *p*-values or confidence intervals were captured. Data extraction was carried out by the first author (RA), with any uncertainties resolved by referring back to the original articles.

## 3. Data Analysis

Data were synthesized narratively by determinant domain and country context. Measures of association (β, r, odds ratios, and risk ratios) were summarized using the most fully adjusted estimates, interpreted by direction and statistical significance (*p*-values < 0.05), and reported with 95% Confidence intervals (95% CI). Meta-analysis was not undertaken due to heterogeneity in study designs and measures.

## 4. Results

### 4.1. Study Characteristics

As shown in Figure 1 (PRISMA flow diagram), a total of 5312 records were identified (5058 through database searches and 254 from Google Scholar). After de-duplication (n = 2374) in Zotero [20], 2938 records were screened by title and abstract, of which 2651 were excluded as clearly not meeting the inclusion criteria. Full texts of 287 reports were sought; 5 could not be retrieved, and 282 were assessed for eligibility. Of these, 228 full-text reports were excluded due to JBI scores < 80% and failure to meet the review criteria (e.g., 117 not related to our study objective, 53 multi-country polls without OIC-specific data, 24 without LE data, 9 with no association estimates, 4 with no OIC-specific data, 3 with major methodological limitations, 1 not a full article, and 16 for other reasons such as only reporting trends or descriptive measures, missing *p*-value, conference paper, etc), and 1 article was ≥80% but was retracted. In the end, only 54 were included in the final systematic review.

Based on the data extraction in Table 1, the 54 included studies were published between 2006 and 2025, with most appearing from 2015 onwards. The majority were time-series or longitudinal ecological/econometric analyses using annual national data (47 studies), with a small number of panel (3 studies) and cross-sectional (4 studies) designs. The evidence covered around two dozen OIC member states and multi-country OIC groupings, with Nigeria (12 studies), Pakistan (5 studies), Oman (5 studies), and Indonesia, Iran, Turkey and Bangladesh (three each) most frequently represented, alongside studies from other individual countries (Malaysia, Saudi Arabia, Kazakhstan, Azerbaijan, Bahrain, Albania, Lebanon, Sudan, Morocco, Palestine) and regional panels of Arab or MENA OIC countries. Study periods typically ranged from 1.25 to 56 years of annual observations. Across the 54 studies, economic/macroeconomic determinants were examined in 44 studies, social/sociodemographic factors in 44, environmental indicators in 33, and healthcare resources or health-system variables in 35, with many analyses including determinants from multiple domains in the same model.

Using the World Bank income classification reflected in the Economy column in Table 2 (low income ≤ $1135; lower-middle $1136–$4495; upper-middle $4496–$13,935; high income ≥ $13,936) [24], the included OIC studies showed a clear income gradient in LE: low-income settings (Sudan, Somalia) consistently reported the lowest LE and the slowest gains, lower-middle-income countries (Nigeria, Pakistan, Bangladesh, Palestine) showed modestly higher LE but strong sensitivity to poverty, education, health spending and environmental pressures, upper-middle-income countries (Indonesia, Iran, Turkey, Kazakhstan, Malaysia, Azerbaijan) generally had higher and more stable LE with increasing influence of health-system capacity and environmental quality, and high-income GCC countries (Oman, Qatar, Saudi Arabia, Bahrain) had the highest LE, where further improvements were linked more to health-system resources, service coverage and sustainability than to income growth alone.

**Table 1:** Quality appraisal of included studies using JBI checklists (≥80% inclusion threshold).

Article	Study	Q1	Q2	Q3	Q4	Q5	Q6	Q7	Q8	Q9	Q10	Q11	Score (%)
Dare et al., 2024 [25]	Longitudinal Time series (observational)	Y	Y	Y	Y	Y	Y	Y	Y	NA	NA	NA	100
Onwube et al., 2021 [26]	Longitudinal Time series (observational)	Y	Y	Y	Y	Y	Y	Y	Y	NA	NA	NA	100
Lawanson & Umar, 2021 [27]	Ecological time-series (country-level)	Y	Y	Y	Y	Y	Y	Y	Y	NA	NA	NA	100
Bahuli et al., 2025 [28]	Time series	Y	Y	Y	Y	Y	Y	Y	Y	NA	NA	NA	100
Popoola & Mohammed, 2024 [29]	Time series econometric study	Y	Y	Y	Y	Y	Y	Y	Y	NA	NA	NA	100
Susanto et al., 2025 [30]	Time series	Y	Y	Y	Y	Y	Y	Y	Y	NA	NA	NA	100
Gilligan & Skrepnek, 2015 [31]	Cross-sectional time series	Y	Y	Y	Y	Y	Y	Y	Y	NA	NA	NA	100
Wirayuda et al., 2023 [32]	Ecological study	Y	Y	Y	Y	Y	Y	Y	Y	NA	NA	NA	100
Ali & Ahmad, 2014 [33]	Time series	Y	Y	Y	Y	Y	Y	Y	Y	NA	NA	NA	100
Wirayuda et al., 2024 [34]	Ecological retrospective study	Y	Y	Y	Y	Y	Y	Y	Y	NA	NA	NA	100
Wirayuda et al., 2025 [35]	Longitudinal ecological study	Y	Y	Y	Y	Y	Y	Y	Y	NA	NA	NA	100
Azam et al., 2022 [36]	Ecological/time-series econometric study	Y	Y	Y	Y	Y	Y	Y	Y	NA	NA	NA	100
ur Rehman et al., 2023 [37]	Time series	Y	Y	Y	Y	Y	Y	Y	Y	NA	NA	NA	100
Sabra, 2022 [38]	Time series	Y	Y	Y	Y	Y	Y	Y	Y	NA	NA	NA	100
Jarallah et al., 2024 [39]	Ecological study/Time series	Y	Y	Y	Y	Y	Y	Y	Y	NA	NA	NA	100
Aziz et al., 2025 [40]	Time series	Y	Y	Y	Y	Y	Y	Y	Y	NA	NA	NA	100
Mohamed, 2020 [41]	Time series	Y	Y	Y	Y	Y	Y	Y	Y	NA	NA	NA	100
Karma, 2023 [42]	Time series	Y	Y	Y	Y	Y	Y	Y	Y	Y	Y	Y	100
Javanshirova, 2024 [43]	Time series	Y	Y	Y	Y	Y	Y	Y	Y	Y	Y	Y	100
Wirayuda et al., 2022 [11]	Time series	Y	Y	Y	Y	Y	Y	Y	Y	Y	Y	Y	100
Xiang et al., 2025 [44]	Time series	Y	Y	Y	Y	Y	Y	Y	Y	Y	Y	Y	100
Panzabekova & Digel, 2020 [45]	Ecological/panel (longitudinal) study	Y	Y	Y	Y	Y	Y	Y	Y	NA	NA	NA	100
Hasan et al., 2023 [46]	Time series econometric study	Y	Y	Y	Y	Y	Y	Y	Y	Y	Y	Y	100
Akintunde et al., 2024 [47]	Time series	Y	Y	Y	Y	Y	Y	Y	Y	Y	Y	Y	100
Audi & Ali, 2016 [48]	Time series	Y	Y	Y	Y	Y	Y	Y	Y	NA	NA	NA	100
Redzwan & Ramli, 2024 [49]	Time series	Y	Y	Y	Y	Y	Y	Y	Y	NA	NA	NA	100
Boutayeb & Serghini, 2006 [50]	Ecological cross-sectional, multi-country comparative study	Y	Y	Y	Y	Y	Y	Y	Y	NA	NA	NA	100
Chan & Kamala Devi, 2015 [51]	Ecological study/Time series	Y	Y	Y	Y	Y	Y	Y	Y	NA	NA	NA	100
Fikri & Mo-hamed, 2024 [52]	Time series econometric study	Y	Y	Y	Y	Y	Y	Y	Y	NA	NA	NA	100
Bala et al., 2025 [53]	Ecological/time series econometric study	Y	Y	Y	Y	Y	Y	Y	Y	NA	NA	NA	100
Senturk & Ali, 2021 [54]	Time series	Y	Y	Y	Y	Y	Y	Y	Y	NA	NA	NA	100
Gulcan, 2020 [55]	Time series	Y	Y	Y	Y	Y	Y	Y	Y	NA	NA	NA	100
Çavmak et al., 2024 [56]	Longitudinal Time series	Y	Y	Y	Y	Y	Y	Y	Y	UC	UC	Y	100
Hamidi et al., 2018 [57]	Ecological Time series	Y	Y	Y	Y	Y	Y	Y	Y	Y	Y	Y	100
Saidmamatov et al., 2024 [58].	Panel data econometric study	Y	Y	Y	Y	Y	Y	Y	Y	NA	NA	NA	100
Kristanto et al., 2019 [59]	Subnational panel regression	Y	Y	Y	Y	Y	Y	Y	Y	NA	NA	NA	100
Pourshahri et al., 2022 [60]	Population-based cross-sectional study	Y	Y	Y	Y	Y	Y	Y	Y	NA	NA	NA	100
Esmaeili et al., 2011 [61]	Cross-sectional (cross-country)	Y	Y	Y	Y	Y	Y	Y	Y	NA	NA	NA	100
Nathaniel & Khan, 2020 [62]	Time series econometric study	Y	Y	Y	Y	Y	Y	Y	Y	NA	NA	NA	100
Agbanike et al., 2019 [63]	Ecological Time series	Y	Y	Y	Y	Y	Y	Y	Y	NA	NA	NA	100
Aalipour et al., 2023 [64]	Time series econometric study	Y	Y	Y	Y	Y	Y	Y	Y	NA	NA	NA	100
Kanat et al., 2023 [65]	Time series econometric study	Y	Y	Y	Y	Y	Y	Y	Y	NA	NA	NA	100
Igbinedion, 2019 [66]	Ecological Time series econometric study	UC	Y	Y	Y	Y	Y	Y	Y	NA	NA	NA	87.5
Okogor, 2022 [67]	Ecological Time series econometric study	Y	Y	Y	Y	UC	Y	Y	Y	NA	NA	NA	87.5
Awan et al., 2024 [68]	Ecological study/Time series	Y	Y	Y	Y	UC	Y	Y	Y	NA	NA	NA	87.5
M. Arafat et al., 2022 [69]	Time series econometric study	Y	Y	Y	Y	UC	Y	Y	Y	NA	NA	NA	87.5
Abbas et al., 2024 [70]	Ecological study/Time series	Y	Y	Y	Y	UC	Y	Y	Y	NA	NA	NA	87.5
Omri et al., 2022 [71]	Ecological study/Time series	UC	Y	Y	Y	Y	Y	Y	Y	NA	NA	NA	87.5
Hussein et al., 2024 [72]	Ecological study/Time series	UC	Y	Y	Y	Y	Y	Y	Y	NA	NA	NA	87.5
Nandi et al., 2023 [73]	Ecological study/Time series	Y	Y	Y	Y	UC	Y	Y	Y	NA	NA	NA	87.5
Setiawan et al., 2023 [74]	Ecological study/Time series	Y	Y	Y	Y	UC	Y	Y	Y	NA	NA	NA	87.5
Ghaedrahmati & Hajilou, 2022 [75]	Ecological study/Time series	Y	Y	Y	Y	UC	Y	Y	Y	NA	NA	NA	87.5
Adeshina et al., 2019 [76]	Ecological study/Time series	Y	Y	Y	Y	Y	UC	Y	Y	NA	NA	NA	87.5
Wirayuda, Jaju, et al., 2022 [77]	Ecological study/Time series	Y	Y	Y	Y	UC	Y	Y	Y	NA	NA	NA	87.5

Note: Y = Yes; N = No; UC = Unclear; NA = Not applicable. For cross-sectional studies (JBI analytical cross-sectional checklist): Q1. Were the criteria for inclusion in the sample clearly defined? Q2. Were the study subjects and the setting described in detail? Q3. Was the exposure measured validly and reliably? Q4. Were objective, standard criteria used for measurement of the condition? Q5. Were confounding factors identified? Q6. Were strategies to deal with confounding factors stated? Q7. Were the outcomes measured validly and reliably? Q8. Was an appropriate statistical analysis used? For cohort studies (JBI cohort checklist): Q1. Were the two groups similar and recruited from the same population? Q2. Were the exposures measured similarly to assign people to both the exposed and unexposed groups? Q3. Was the exposure measured validly and reliably? Q4. Were confounding factors identified? Q5. Were strategies to deal with confounding factors stated? Q6. Were the groups/participants free of the outcome at the start of the study (or at the moment of exposure)? Q7. Were the outcomes measured validly and reliably? Q8. Was the follow-up time reported and sufficient to be long enough for outcomes to occur? Q9. Was follow-up complete, and if not, were the reasons for loss to follow-up described and explored? Q10. Were strategies to address incomplete follow-up utilized? Q11. Was an appropriate statistical analysis used?

**Table 2:** Characteristics of studies on determinants of life expectancy in OIC member countries included in the systematic review.

Authors	Year of Publication	Study Design	Country	Economy (World Bank)	Study Period (Years)	Study Population	Sample Size	Determinant/ Factors Category	Determinants/Factors	**Measure of Association**
Dare et al., 2024 [25]	2024	Longitudinal Time series (observational)	Nigeria	Lower-Middle income economies ($1136 to $4495)	2012–2022 (11)	Nigerian	11 annual observations	Social Environmental Economic/finance (globalization & green finance)	Trade openness: (TROP) Net foreign domestic product: (NFDI) Net foreign portfolio investment: (NFPI) Green bonds (GREB) Renewable energy investment (RENI) Credit to agriculture (CRAG) Gross domestic product (GRDP)	Crude TROP r = −0.48861 NFDI r = −0.82638 NFPI r = 0.68488 GREB r = −0.54214 RENI r = 0.03774 CRAG r = 0.44827 GRDP r = 0.74412 Adjusted aTROP β = −0.129393 (*p* = 0.2772) aNFDI β = −0.001728 (*p* = 0.1427) aNFPI β = 1.35 × 10^−5^ (*p* = 0.0002) aGREB β = −0.000289 (*p* = 0.6602) aRENI β = −0.611983 (*p* = 0.2002) aCRAG β = 1.97 × 10^−6^ (*p* = 0.0032) aGRDP β = 0.000861 (*p* = 0.2079)
Onwube et al., 2021 [26]	2021	Longitudinal Time series (observational)	Nigeria	Lower-Middle income economies ($1136 to $4495)	1981–2017 (37)	Nigerian	37 annual observations	Social Economic (macroeconomic determinants)	GDP per capita (constant 2010 US$): RGDPPC Inflation rate, consumer prices (annual %): INFR Imports of goods and services (per capita constant 2010 US$): Imports Household final consumption expenditure (per capita constant 2010 US$): HCExp General government final consumption expenditure (Per capita constant 2010 US$): GCExp Official exchange rate (LCU per US$, period average): EXR	Adjusted RGDPPC β = 0.100954 (*p* = 0.0009) INFR β = −0.034493 (*p* = 0.0001) Import β = −0.068840 (*p* < 0.001) HCE β = 0.021552 (*p* = 0.1667) GCE β = −0.024102 (*p* = 0.0004) EXR β = 0.017021 (*p* < 0.001)
Lawanson & Umar, 2021 [27]	2021	Ecological time series (country-level)	Nigeria	Lower-Middle income economies ($1136 to $4495)	1980–2018 (39)	Nigerian	38 annual observations	Health status Education Economic Poverty Governance-finance	Per capita gross domestic product: PCGDP Poverty: Poverty headcount: PHC Poverty gap: PGAP Squared poverty gap: SPGAP	PCGDP β = 0.140123, *p* = 0.0001 Poverty: PHC: β = −0.1672, *p* = 0.0006 PGAP: β = −0.1401, *p* = 0.0011 SPGAP: β = −0.1223, *p* = 0.0026
Bahuli et al., 2025 [28]	2025	Time series	Nigeria	Lower-Middle income economies ($1136 to $4495)	1990–2022 (33)	Nigerian	33 annual observations	Environmental Economic Demographic Investment/Globalization	CO_2_ emissions: CO_2_ GDP per capita: GDP Population growth rate: POP Foreign direct investment inflow: FDI	CO_2_ β = −2.185889 (*p* = 0.3629) FDI β = −0.070688 (*p* = 0.0644) GDP β = −1.19 × 10^−5^ (*p* = 0.9992) POP β = 5.085600 (*p* = 0.0001)
Popoola & Mohammed, 2024 [29]	2024	Time series econometric study	Nigeria	Lower-Middle income economies ($1136 to $4495)	1986–2022 (37)	Nigerian	37 annual observations	Macroeconomic Socio-demographic	Domestic debt: DD External debt: ED Real GDP per capita: RGDP Household consumption expenditure: CEH Population growth rate: PGR	Adjusted DD β = −0.3520, *p* = 0.2411 ED β = −0.0106, *p* = 0.0902 RGDP β = 0.4132, *p* = 0.0150 CEH β = −0.2458, *p* = 0.2501 PGR β = 4.1052, *p* = 0.0006
Susanto et al., 2025 [30]	2025	Time series	Indonesia	Upper-Middle-income economies ($4496 to $13,935)	2010–2018 (9)	46 OIC member states: Benin, Burkina Faso, Chad, Djibouti, Gambia, Guinea, Guinea-Bissau, Comoros, Mali, Mauritania, Mozambique, Niger, Senegal, Sierra Leone, Somalia, Sudan, Togo, Uganda, Afghanistan, Bangladesh, Yemen, Indonesia, Kazakhstan, Kyrgyzstan, Azerbaijan, Bahrain, Brunei Darussalam, Lebanon, Maldives, Malaysia, Oman, Pakistan, Syria, Tajikistan, Turkmenistan, Uzbekistan and Jordan, Albania, Turkey, Guyana, Suriname, Cameroon, Morocco, Egypt, Ivory Coast and Tunisia, Saudi Arabia, United Arab Emirates, Iraq, Kuwait, Iran, Qatar, Nigeria, Algeria, Libya, Gabon	46 countries × 9 years = 414 country-years	Social Healthcare resources Behavioural	GDP per capita, log: LN_GDP Health expenditure %GDP: HEXP Mean years of schooling: SCH Income inequality: GINI Unemployment rate: UNEP Smoking prevalence: SMOKE	Adjusted LN_GDP β = 6.019235 (*p* = 0.0000) HEXP β = 0.132586 (*p* = 0.0400) SCH β = 0.575393 (*p* = 0.0000) GINI β = 0.012648 (*p* = 0.5264) UNEP β = −0.009166 (*p* = 0.8055) SMOKE β = −0.220921 (*p* = 0.0000)
Gilligan & Skrepnek, 2015 [31]	2015	Cross-sectional time series	Eastern Mediterranean Region (21 countries)	Mixed	1995–2010 (16)	21 countries: Afghanistan, Kuwait, Saudi Arabia, Bahrain, Lebanon, Somalia, Djibouti Libyan, Sudan, Egypt, Morocco, Syria, Iran, Oman, Tunisia, Iraq, Pakistan, United Arab Emirates, Jordan, Qatar, Yemen	21 countries (panel across 1995–2010; up to ~336 country-years)	Social/Economic Healthcare resources Environmental/living conditions	GDP per capita: GDP Health expenditure: HE Physician density: PHYS Vaccination average: VACC Adult literacy: LIT Safe water access: WATER Urbanization: URBAN Undernourishment: UNOURISH	Adjusted GDP β = 0.0229 (*p* = 0.011) HE β = −0.0049 (*p* = 0.387) PHYS β = 0.0079 (*p* = 0.266) VACC β = 0.0018 (*p* = 0.026) LIT β = 0.0001 (*p* = 0.889) WATER β = 0.0012 (*p* = 0.097) URBAN β = 0.0021 (*p* = 0.026) UNOURISH β = −0.0009 (*p* = 0.520)
Wirayuda et al., 2023 [32]	2023	Ecological study	Oman	High-income economies ($13,935 or more)	1990–2020 (31)	Country-level (Oman & Qatar)	31 annual observations per country (1990–2020 = 31 years)	Macroeconomic (ME) Sociodemographic (SD) Health Status & Resources (HSR)	Gross National Income (GNI) per capita: GNIpc Employment to population ratio: Employment Oil production: Fuel Pre-Primary School Enrolment: PPSE Primary School Enrolment: PSE Secondary School Enrolment: SSE Diphtheria, Pertussis, and Tetanus (DPT) Immunization: DPTI Measles Immunization: MI Food production index: Food	Oman HSR → LE (direct): β = 0.839 (95% CI 0.717–0.894) SD → LE (indirect via HSR): β = 0.653 (95% CI 0.450–0.754) ME → LE (indirect via SD and HSR): β = 0.602 (95% CI 0.407–0.709) Qatar HSR → LE (direct): β = 0.904 (95% CI 0.707–0.956) SD → LE (indirect via HSR): β = 0.759 (95% CI 0.550–0.885) ME → LE (indirect via SD and HSR): β = 0.676 (95% CI 0.438–0.845)
Ali & Ahmad, 2014 [33]	2014	Time series	Oman	High-income economies ($13,935 or more)	1970–2012 (43)	Omanis	43 annual observations	Social/Economic Environmental Food/Nutrition	Food production index: FI School enrolment-primary: ED/EE Inflation: INF Population growth: POPg GDP per capita growth: PCg CO_2_ emissions: CO_2_	Adjusted FI β = 0.115652 (*p* = 0.000) INF β = −0.005085 (*p* = 0.133) POPg β = −0.245641 (*p* = 0.027) Ee β = 0.154537 (*p* = 0.000) PCg β = −0.001035 (*p* = 0.961) CO_2_ β = 0.216793 (*p* = 0.736)
Wirayuda et al., 2024 [34]	2024	Ecological retrospective study	Oman	Mixed	1980–2020 (41)	Omanis and Indonesians	41 annual observations per country	Macroeconomic: ME Sociodemographic: SD Health Status–Resources: HSR	ME: GDP: Gross Domestic Product per capita CI: Capital Investment EP: Electricity Production SD: PrE: Pre-Primary School Enrolment SE: Secondary School Enrolment TE: Tertiary School Enrolment HSR: DPT: Diphtheria, Pertussis, and Tetanus Immunization MI: Measles Immunization FPI: Food production index	Indonesia ME → LE (total): β = 0.737 (95% CI 0.527–0.904) SD → LE (total): β = 0.675 (95% CI 0.493–0.824) (indirect via HSR) HSR → LE (direct): β = 0.823 (95% CI 0.653–0.946) Oman ME → LE (total): β = 0.848 (95% CI 0.784–0.899) SD → LE (total): β = 0.755 (95% CI 0.613–0.918) HSR → LE (direct): β = 0.335 (95% CI 0.047–0.525
Wirayuda et al., 2025 [35]	2025	Longitudinal ecological study	Oman	High-income economies ($13,935 or more)	1990–2020 (31)	GCC Countries: Bahrain, Kuwait, Oman, Qatar, Saudi Arabia, UAE	6 countries × 31 years = 186 country-years	Macroeconomic: ME Sociodemographic: SD Health Resources: HR	ME: ME1: Gross Domestic Product (GDP) per capita ME2: Electricity Production SD: SD1: Pre-primary School Enrolment SD2: Secondary School Enrolment HR: HR1: Diphtheria, Pertussis, and Tetanus Immunization HR2: Measles Immunization HR3: Food production index	Crude Pooled (all GCC): ME-LE r = 0.9000 *; SD-LE r = 0.7640 *; HR-LE r = 0.8940 * Bahrain: ME-LE r = 0.9699 *; SD-LE r = 0.9742 *; HR-LE r = 0.9389 * UAE: ME-LE r = 0.9550 *; SD-LE r = 0.9394 *; HR-LE r = 0.8528 * Kuwait: ME-LE r = 0.7856 *; SD-LE r = 0.4240 *; HR-LE r = 0.8777 * Oman: ME-LE r = 0.9535 *; SD-LE r = 0.9364 *; HR-LE r = 0.8387 * Qatar: ME-LE r = 0.9230 *; SD-LE r = 0.9299 *; HR-LE r = 0.9497 * Saudi Arabia: ME-LE r = 0.8223 *; SD-LE r = 0.4430 *; HR-LE r = 0.8754 * Adjusted Pooled “general” SEM (all GCC countries combined) HR → LE: β = 0.468 (*p* < 0.001) ME → LE: β = 0.510 (*p* < 0.001) Country-specific total effects on LE (separate for each GCC/OIC country) Bahrain: HR → LE 0.3427 *, ME → LE 0.6747 *, SD → LE 0.9530 * UAE (Emirate): HR → LE 0.3746 *, ME → LE 0.6831 *, SD → LE 0.9258 * Kuwait: HR → LE 0.5858 (ns), ME → LE 0.2942 (ns), SD → LE 0.5693 * Oman: HR → LE 0.2213 (ns), ME → LE 0.7225 *, SD → LE 0.8373 * Qatar: HR → LE 0.5709 *, ME → LE 0.4518 *, SD → LE 0.9024 * Saudi Arabia: HR → LE 0.6052 *, ME → LE 0.3270 *, SD → LE 0.2572 (ns) Significant values are presented with an asterisk (*) at a 5% level.
Azam & Adeleye, 2022 [36]	2022	Ecological/time series econometric study	Pakistan	Lower-Middle income economies ($1136 to $4495)	1975–2020 (46)	Pakistanis	46 annual observations	Environmental Economic Food/agriculture Demographic Health system Education	CO_2_ Carbon emissions PCI Per capita income FPI Food production index POPG Population growth BR Birth rate DR Death rate IMF Infant mortality rate The Health expenditure INF Inflation EDU Education	ARDL CO_2_ emissions: β = −0.046395 (*p* = 0.0007) Per capita income: β = 0.001144 (*p* = 0.8812) Food production index: β = −0.010727 (*p* = 0.0890) Population growth: β = 0.008288 (*p* = 0.0512) Birth rate: β = 0.466607 (*p* = 0.0000) Death rate: β = −0.911756 (*p* = 0.0000) Infant mortality rate: β = 0.178382 (*p* = 0.0091) Health expenditure: β = 0.000215 (*p* = 0.0295) Inflation: β = −0.002072 (*p* = 0.0354) Education: β = 0.02002 (*p* = 0.0514) Robustness checks: FMOLS & DOLS FMOLS: CO_2_: β = −0.007595 (*p* = 0.0530) Per capita income: β = 0.024526 (*p* = 0.0000) Food production index: β = −0.006692 (*p* = 0.0302) Population growth: β = 0.00634 (*p* = 0.0000) Birth rate: β = 0.167738 (*p* = 0.0000) Death rate: β = −0.342165 (*p* = 0.0000) Infant mortality rate: β = 0.029426 (*p* = 0.0000) Health expenditure: β = 0.001703 (*p* = 0.0000) Inflation: β = −0.000541 (*p* = 0.0005) Education: β = 0.002470 (*p* = 0.0024) DOLS: CO_2_: β = −0.013032 (*p* = 0.0289) Per capita income: β = 0.019516 (*p* = 0.0532) Food production index: β = −0.014553 (*p* = 0.0209) Population growth: β = 0.002284 (*p* = 0.0949) Birth rate: β = 0.165772 (*p* = 0.0035) Death rate: β = 0.378559 (*p* = 0.0004) Infant mortality rate: β = 0.015604 (*p* = 0.0778) Health expenditure: β = 0.000372 (*p* = 0.4076) Inflation: β = −0.001049 (*p* = 0.0347) Education: β = 0.001664 (*p* = 0.0501)
ur Rehman et al., 2023 [37]	2023	Time series	Pakistan	Lower-Middle income economies ($1136 to $4495)	1980–2020 (41)	Pakistanis	41 annual observations	Social (income inequality) Economic (income) Health resources	GINI: income inequality GDPPC/GPC: GDP per capita HE: Health expenditure	Adjusted GINI β = −0.25060 (*p* = 0.0044) HE β = −0.92628 (*p* = 0.3703) GDPPC β = 0.02238 (*p* = 0.0000)
Sabra, 2022 [38]	2022	Time series	Palestine	Lower-Middle income economies ($1136 to $4495)	2000–2019 (20)	Algeria, Egypt, Lebanon, Morocco, and Tunisia	6 countries × 20 years = 120 country-years	Economic Environmental Demographic Health resources	Total Population: POP Gross Domestic Product: GDP Current health expenditure per capita: CHE Fertility rate, births per woman: Fertility CO_2_ emission: CO	POP β = 0.003 (*p* < 0.01) CHE β = 0.002 (*p* < 0.01) GDP β = −0.0012 (*p* < 0.01) CO_2_ β = −0.003 (*p* < 0.01) Fertility β = −0.005 (*p* < 0.01)
Jarallah et al., 2024 [39]	2024	Ecological study/Time series	Qatar	High-income economies ($13,935 or more)	2000–2020 (21)	GCC countries (Bahrain, Kuwait, Oman, Qatar, Saudi Arabia, UAE)	126 country-year observations (6 countries × 21 years)	Environmental Socioeconomic Health resources Technology	Ecological footprint deficit: ECOLDEF Urbanization: URB Unemployment: UNEMP Health expenditure per capita: CURHEPC GDP deflator/inflation proxy: GDPDEF Technological Achievement Index: TAI A composite index of mobile, fixed telephone, and internet subscriptions: ICTINDEX Carbon dioxide emission: CO_2_	Crude URB: r = 0.3934 UNEMP: r = −0.6023 CO_2_: r = −0.2287 ICTINDEX: r = 0.5596 CURHEPC: r = 0.4604 GDPDEF: r = −0.2267 ECOLDEF: r = −0.6269 TAI: r = 0.6413 Adjusted Pooled (GCC) adjusted associations with LE UNEMP: β = −7.0681 (*p* = 0.011) CURHEPC: β = −0.0083 (*p* = 0.041) GDPDEF: β = 0.0143 (*p* = 0.669) (ns) ECOLDEF: β = −2.5654 (*p* = 0.034) TAI: β = 88.9262 (*p* = 0.015) Country-specific adjusted effects Bahrain URB: β = −0.4393 (*p* = 0.0000); UNEMP: β = −0.0108 (*p* = 0.2320); CURHEPC: β = 0.0000 (*p* = 0.0670); GDPDEF: β = −0.0001 (*p* = 0.4500); ECOLDEF: β = −0.0028 (*p* = 0.0010); TAI: β = 0.1734 (*p* = 0.0100) Kuwait URB: β = 0.0209 (*p* = 0.4760); UNEMP: β = 0.0668 (*p* = 0.0030);m CURHEPC: β = −0.0001 (*p* = 0.0680); GDPDEF: β = −0.0013 (*p* = 0.0000); ECOLDEF: β = 0.0107 (*p* = 0.1950); TAI: β = 1.0148 (*p* = 0.0010) Oman URB: β = −0.0170 (*p* = 0.4100); UNEMP: β = −0.0481 (*p* = 0.0080); CURHEPC: β = −0.0001 (*p* = 0.0780); GDPDEF: β = −0.0002 (*p* = 0.3460); ECOLDEF: β = −0.0144 (*p* = 0.0300); TAI: β = −1.7191 (*p* = 0.0060) Qatar URB: β = 0.3385 (*p* = 0.0000); UNEMP: β = 0.0303 (*p* = 0.1400); CURHEPC: β = 0.0000 (*p* = 0.0170); GDPDEF: β = 0.0000 (*p* = 0.7230); ECOLDEF: β = 0.0028 (*p* = 0.2220); TAI: β = 0.0250 (*p* = 0.8880) Saudi Arabia URB: β = 6.5084 (*p* = 0.0110); UNEMP: β = 0.0644 (*p* = 0.0000); CURHEPC: β = 0.0000 (*p* = 0.9560); GDPDEF: β = −0.0007 (*p* = 0.0540); ECOLDEF: β = 0.0116 (*p* = 0.3700); TAI: β = 0.5573 (*p* = 0.1940) United Arab Emirates URB: β = 0.2862 (*p* = 0.0000); UNEMP: β = 0.0025 (*p* = 0.5440); CURHEPC: β = 0.0000 (*p* = 0.6740); GDPDEF: β = 0.0000 (*p* = 0.8090); ECOLDEF: β = 0.0020 (*p* = 0.2690); TAI: β = −0.0194 (*p* = 0.8390)
Aziz et al., 2025 [40]	2025	Time series	Saudi Arabia	High-income economies ($13,935 or more)	1980–2020 (41)	Saudi Arabia	41 annual observations	Environmental Economic Health resources	Economic growth: EG Health expenditure: HE Ecological footprint: EFP CO_2_ emissions: Carbon Particulate matter 2.5: PM2.5	Crude: EG (GDP): r = 0.716 HE: r = 0.792 EFP: r = 0.507 Carbon (CO_2_): r = 0.654 PM2.5: r = 0.542 Adjusted (pre-Vision 2030) EG β = 0.523 (*p* < 0.001) HE β = 0.671 (*p* < 0.005) EFP β = 1.204 (*p* > 0.010) Carbon β = 0.631(*p* < 0.001) PM2.5 β = 0.095 (*p* < 0.005) (post-Vision 2030) EG β = 0.523 (*p* < 0.005) HE β = 0.481(*p* < 0.001) EFP β = 1.190 (*p* > 0.010) Carbon β = 0.501(*p* < 0.005) PM2.5 β = 0.084 (*p* < 0.005)
Mohamed, 2020 [41]	2020	Time series	Sudan	Low-income economies ($1135 or less)	1970–2017 (48)	Sudanese	48 annual observations	Economic Social Environmental	GDP (outcome) Investment: INV Youth unemployment: YUN Life expectancy: LE Education: EDU Access to sanitation: ASF Access to safe water: ASW Access to electricity: ELC CO_2_ per capita: CO_2_P Trade openness: TOP	Crude GDP: r = 0.90 INV: r = 0.22 YUN: r = −0.29 EDU: r = 0.97 ASF: r = −0.48 ASW: r = 0.91 ELC: r = 0.93 CO_2_P: r = 0.44 TOP: r = 0.10 Adjusted GDP β = 19.44 (*p* < 0.000)
Karma, 2023 [42]	2023	Time series	Albania	Upper-Middle-income economies ($4496 to $13,935)	2000–2019 (20)	Southeastern Europe (SEE), including Albania	20 annual observations	Economic Social Environmental	Health expenditure: HE GDP per capita: GDP Out-of-pocket health expenditure %: OPEH Education: EDU Marriages/1000: MS Fertility rate: FER CO_2_ per capita: CO_2_ Urban population %: URB	HE β = 0.02 EDU β = −0.01 GDP β = 0.03 MS = β = 0.03 * OPEH β = −0.04 FER β = −0.02 CO_2_ β = 0.01 URB β = −0.04 (* indicates *p* < 0.50; others not significant)
Javanshirova, 2024 [43]	2024	Time series	Azerbaijan	Upper-Middle-income economies ($4496 to $13,935)	1974–2022 (49)	Azerbaijanis/Azeris	49 annual observations	Environmental	Air pollution/climate: CO_2_	Crude CO_2_ (r = −0.8102) Adjusted CO_2_ β = −0.1577 (*p* < 0.001)
Wirayuda et al., 2022 [11]	2022	Time series	Bahrain	High-income economies ($13,935 or more)	1971–2020 (50)	Bahrainis	50 annual observations	Macroeconomic: ME Sociodemographic: SD Health Status & Resources: HSR	Pre-primary education: PPE Primary education: PE Tertiary education: TE Gross Domestic Product: GDP GDP per capita: GDPpc Fossil-fuel electricity: FF Measles immunization: MI DPT immunization: DPTI	Adjusted PLS-SEM path coefficients ME → LE β = 0.463 (*p* < 0.00) HSR → LE β = 0.595 (*p* < 0.001) ME → SD → HSR → LE indirect β = 0.488 (*p* < 0.001) SD → HSR → LE indirect β = 0.496 (*p* < 0.001) ME → SD β = 0.984 (*p* < 0.001) SD → HSR β = 0.835 (*p* < 0.001) ME total effect on LE β = 0.95 (*p* < 0.001)
Xiang et al., 2025 [44]	2025	Time series	Bangladesh	Lower-Middle income economies ($1136 to $4495)	2000–2022 (23)	Bangladeshis	23 annual observations	Healthcare resources Economic Financial Demographic Shock	Public Health Expenditure (% of GDP): PHE GDP per Capita (current US$): GDPPC Domestic Credit to Private Sector (% GDP): DC Population Growth Rate (%): POPGR	Non-adjusted model (no COVID) PHE (L0): β = −0.042, *p* > 0.10 PHE (L1): β = 0.0252, *p* > 0.10 GDPPC (L0): β = 0.0002, *p* > 0.10 GDPPC (L1): β = −0.0011, *p* < 0.01 DC (L0): β = 0.0603, *p* < 0.05 POPGR (L0): β = −1.5289, *p* > 0.10 POPGR (L1): β = 1.0497, *p* > 0.10 Adjusted model includes COVID PHE (L0): β = −0.038 (*p* > 0.10) PHE (L1): β = 0.01 (*p* > 0.10) GDPPC (L0): β = 0.0003 (*p* > 0.10) GDPPC (L1): β = −0.0012 (*p* < 0.01) DC (L0): β = 0.055 (*p* < 0.05) POPGR (L0): β = −1.40 (*p* > 0.10) POPGR (L1): β = 0.964 (*p* > 0.10) COVID dummy (2020–22): β = −0.213 (*p* < 0.10)
Panzabekova & Digel, 2020 [45]	2020	Ecological/panel (longitudinal) study	Kazakhstan	Upper-Middle-income economies ($4496 to $13,935)	2001–2018 (18)	Kazakh/Kazakhstanis	18 annual observations	Economic Demographic/social Health system Medical/morbidity Crime	Nominal monetary income: NMI Subsistence minimum: SM Income-to-subsistence ratio: NISM Poverty: *p* Unemployment: U Divorces per 1000 marriages: DpM Health workers: HW Cancer morbidity: CMR Blood diseases: BD Substance-induced mental disorders: MD Circulatory system diseases: CSD respiratory diseases: RD Crime rate: CR	Crude (regional base) Akmola: DpM 0.958; MD −0.954; U −0.928; SM 0.914; NMI 0.904; *p* −0.888; NISM 0.867; CR 0.831; RD 0.743; CSD 0.713 Aktobe: NMI 0.977; SM 0.973; *p* −0.959; U −0.955; NISM 0.916; RD −0.909; MD −0.832; HW 0.818; CMR −0.784 Almaty region: MD −0.907; SM 0.885; HW 0.884; DpM 0.884; NMI 0.883; *p* −0.879; CR 0.854; NISM 0.849; CSD 0.789; CR 0.768 Atyrau: NMI 0.953; SM 0.948; *p* −0.936; U −0.893; HW 0.885; NISM 0.879; DpM 0.854; MD −0.802 West Kazakhstan region: NMI 0.964; SM 0.949; *p* −0.938; U −0.930; MD −0.892; NISM 0.865; CR 0.865; DpM 0.826; CSD 0.770 Jambyl: CMR 0.924; SM 0.907; NMI 0.888; DpM 0.866; U −0.861; RD 0.856; HW 0.845; *p* −0.825; NISM 0.800; CR 0.700 Karaganda: MD −0.921; SM 0.918; DpM 0.903; NMI 0.902; *p* −0.890; U −0.872; CSD 0.826; BD −0.815; BD −0.776; CR 0.697 Kostanay: MD −0.920; DpM 0.902; SM 0.876; U −0.874; NMI 0.872; *p* −0.829; CMR 0.819; NISM 0.803 Kyzylorda: DpM 0.937; *p* −0.910; SM 0.907; U −0.898; NMI 0.894; MD −0.847; HW 0.831; NISM 0.824; RD 0.854; CSD 0.737 Mangistau: U −0.979; MD −0.978; SM 0.975; NMI 0.969; HW 0.948; *p* −0.945; *p* −0.895; CSD 0.869; RD 0.808; DpM 0.843; CR 0.765 Pavlodar: MD −0.971; NMI 0.929; CMR 0.928; SM 0.920; U −0.912; NISM 0.908; MD −0.842; CMR 0.836 Turkestan + Shymkent: SM 0.927; NMI 0.903; DpM 0.887; *p* −0.877; U −0.871; NISM 0.782; CSD 0.765; MD −0.763; DpM 0.725 East Kazakhstan: CSD 0.965; U −0.937; NMI 0.907; SM 0.906; NISM 0.882; BD −0.873; *p* −0.862; MD −0.739 Nur-Sultan: NMI 0.978; HW 0.975; SM 0.974; U −0.960; *p* −0.852; CMR 0.827; CR 0.699; CSD 0.698 City of Almaty: SM 0.927; NMI 0.918; U −0.911; HW 0.894; *p* −0.843; CSD 0.827; CR 0.796; NISM 0.757 Adjusted Akmola: β0 = 3.69, DpM β1 = 0.11, MD β2 = −0.03 Aktobe: β0 = 3.5, NMI β1 = 0.05, SM β2 = 0.02 Almaty region: β0 = 3.9, MD β1 = −0.04, DpM β2 = 0.80 Atyrau: β0 = 3.36, NMI β1 = 0.041, DpM β2 = 0.074 West Kazakhstan: β0 = 3.38, NMI β1 = 0.04, CR β2 = 0.075 Jambyl: β0 = 3.59, SM β1 = 0.03, DpM β2 = 0.065 Karaganda: β0 = 4, MD β1 = −0.03, SM β2 = 0.04 Kostanay: β0 = 3.6, MD β1 = −0.04, DpM β2 = 0.13 Kyzylorda: β0 = 3.8, DpM β1 = 0.08, U β2 = −0.04 Mangistau: β0 = 4.64, U β1 = −0.09, MD β2 = −0.04 Pavlodar: β0 = 4.09, MD β1 = −0.035, DpM β2 = 0.056 Northern Kazakhstan: β0 = 3.62, DpM β1 = 0.12, MD β2 = −0.028 Turkestan + Shymkent: β0 = 3.66, SM β1 = 0.13, NMI β2 = −0.06 East Kazakhstan: β0 = 3.83, CSD β1 = 0.12, BD β2 = −0.07 Nur-Sultan (one-factor model): β0 = 3.83, NMI β1 = 0.039 (no X2) City of Almaty: β0 = 2.73, SM β1 = 0.039, HW β2 = 0.012
Hasan et al., 2023 [46]	2023	Time series econometric study	Bangladesh	Lower-Middle income economies ($1136 to $4495)	1990–2019 (30)	Bangladeshis	30 annual observations	Environmental Social/Health Social/Education Technology/Innovation	CO_2_ emissions: lnCO_2_ Secondary school enrolment: lnEDU Total patents/innovation proxy: lnTI	Adjusted GDP β = 4.22, *p* < 0.05
Akintunde et al., 2024 [47]	2024	Ecological-time series	Nigeria	Lower-Middle income economies ($1136 to $4495)	1980–2020 (41)	Nigerians	41 annual observations	Environmental Socioeconomic Economic Healthcare resources	CO_2_ emissions Income inequality: Gini/INC GDP per capita: LGDPC Govt expenditure on health: LGEXH Unemployment: UNEM Gross capital formation: LGCF	CO_2_ emissions: β = −45.0359, *p* = 0.0066 Gini/INC: β = −0.1946, *p* = 0.0293 LGDPC: β = 0.0027, *p* = 0.0847 LGEXH: β = −0.0789, *p* = 0.0659 UNEM: β = −9.4608, *p* = 0.0680 LGCF: β = 0.0023, *p* = 0.1929
Audi & Ali, 2016 [48]	2017	Time series	Lebanon	Lower-Middle income economies ($1136 to $4495)	1971–2014 (42)	Lebanese	42 observations	Socioeconomic Environmental Education Economic Demographic	Availability of food index: FOOD CO_2_ emissions: CO_2_ Secondary school enrolment: SSE GDP per capita: GDPPC Population growth: POPG	FOOD: β = 0.056465 (*p* = 0.0000) CO_2_: β = −1.072773 (*p* = 0.0024) SSE: β = 2.38 × 10^−5^ (*p* = 0.0169) GDPP: β = 0.001062 (*p* = 0.0000) POPG: β = 0.285793 (*p* = 0.0163)
Redzwan & Ramli, 2024 [49]	2024	Time series	Malaysia	Upper-Middle-income economies ($4496 to $13,935)	1997–2021 (25)	Malaysians	25 annual observations	Environmental Health resources Economic	CO_2_ emissions per capita: lnCO_2_ Health expenditure per capita: lnHE GDP per capita: lnGDP	HE: β = −0.2229 (*p* = 0.6609) GDP: β = 0.26132 (*p* = 0.6282) CO_2_: β = 0.0227 (*p* = 0.8438)
Boutayeb & Serghini, 2006 [50]	2006	Ecological cross-sectional, multi-country comparative study	19 Arab countries (all OIC): Algeria, Bahrain, Comoros, Djibouti, Egypt, Jordan, Kuwait, Lebanon, Libya, Mauritania, Morocco, Oman, Qatar, Saudi Arabia, Sudan, Syria, Tunisia, UAE, Yemen	Mixed	Mixed indicator years (mainly 1990–2003: LEB 2002; IMR 2002; literacy 2002; enrolment 2001/02; physicians 1990–2003)	Country-level indicators	19 countries (Iraq, Palestine, and Somalia excluded due to missing data)	Health outcomes; Health services Maternal/child health Nutrition Education	Infant Mortality Rate per 1000 live births 2002: IMR Maternal Mortality Ratio per 100,000–2000: MMR Expectation of lost healthy male/female 2002: ELHf/ELHm Delivery attended by skilled attendant 1996 (%): DASA Pregnant women who received prenatal care in 1996 (%): PWRP Children underweight % of <5 years old 1995–2002: CUW Physicians Per 100,000 People 1990–2003: PPP Literacy male/female (%) 2002: Lm/Lf Enrolment male/female (%) 2002: Enm/Enf	Female life expectancy at birth (LEBf) ELHf: r = −0.59 ELHm: r = −0.65 MMR: r = −0.94 DASA: r = 0.43 PWRP: r = 0.49 CUW: r = −0.60 IMR: r = −0.95 PPP: r = 0.75 Lm: r = 0.65 Lf: r = 0.68 Enm: r = 0.81 Enf: r = 0.87 Male life expectancy at birth (LEBm) ELHf: r = −0.56 ELHm: r = −0.63 MMR: r = −0.94 DASA: r = 0.42 PWRP: r = 0.47 CUW: r = −0.59 IMR: r = −0.95 PPP: r = 0.74 Lm: r = 0.67 Lf: r = 0.69 Enm: r = 0.80 Enf: r = 0.85
Chan & Kamala Devi, 2015 [51]	2015	Ecological study/Time series	Malaysia	Upper-Middle-income economies ($4496 to $13,935)	1980–2008 (29)	Malaysians	29 annual observations	Socioeconomic Demographic Health resources	Gross national income per capita: GDP Inflation rate: IR Literacy rate: LR Tuberculosis deaths/100k: Tuberculosis Doctors/10k: Doctors Per-capita govt health expenditure: Expenditure	Crude Doctor: r = 0.75 *p* < 0.05) Expenditure: r = 0.81 (*p* < 0.05) LR: r = 0.69 (*p* < 0.05) Tuberculosis: r = −0.71 (*p* < 0.05) IR: r = 0.84 (*p* < 0.05) GDP: r = 0.68 (*p* < 0.05) Adjusted Direct effect on LE: Health resources → LE β = 0.47 (*p* < 0.05) Indirect structure (predictors of “Health resources”) Socioeconomic status → Health resources β = 0.57 (*p* < 0.05) Demographic → Health resources β = 0.56, (*p* < 0.05) Socioeconomic status → Demographic β = 0.58, (*p* < 0.05)
Fikri & Mohamed, 2024 [52]	2024	Time series econometric study	Morocco	Lower-Middle income economies ($1136 to $4495)	2000–2022 (23)	Moroccans	23 annual observations	Health/human capital Education/human capital Labour market	Life expectancy at birth: LE School enrolment, tertiary (% gross): SET Labour force participation rate (% ages 15+): LAB Gross capital production: GDP (outcome)	Coefficients with GDP per capita (log) as outcome: LE (current) β = 10.84694 (*p* = 0.0183) LE (lag 1) β = −3.86640 (*p* = 0.2001)
Bala et al., 2025 [53]	2025	Ecological/time series econometric study	Nigeria	Lower-Middle income economies ($1136 to $4495)	1992–2021 (30)	Nigerian	30 annual observations	Macroeconomic Environment Health status Education/socio-demographic	Gross domestic production: GDP Environment sustainability: ENV Mortality rate: MOR Literacy rate: LIT	Adjusted GDP: β = 0.047, *p* = 0.225 (not significant) ENV: β = 0.161, *p* < 0.001 MOR: β = −1.844, *p* = 0.010 LIT: β = 0.232, *p* = 0.022
Senturk & Ali, 2021 [54]	2021	Time series	Turkey	Upper-Middle-income economies ($4496 to $13,935)	1971–2017 (47)	Turks	47 annual observations	Education Environment Economic/purchasing power Economic development Demographic	Education (SSE): Secondary enrolment (overall) Environment (SUS): CO_2_ emissions (environmental degradation) Economic/purchasing power (INF): Inflation Economic development (ECOD): GDP per capita growth Demographic (PG): Population growth	Crude SSE r = 0.989707 (*p* < 0.001) SUS r = 0.992322 (*p* < 0.001) INF r = −0.215419 (*p* > 0.005) ECOD r = 0.951445 (*p* < 0.001) PG r = −0.92861 (*p* < 0.001) Adjusted SSE β = 0.176652 (*p* < 0.001) SUS β = −0.008789 (*p* > 0.005) INF β = 0.000641(*p* > 0.005) ECOD β = −0.135435(*p* > 0.005) PG β = 0.004655 (*p* > 0.005)
Gulcan, 2020 [55]	2020	Time series	Turkey	Upper-Middle-income economies ($4496 to $13,935)	1975–2014 (40)	Turks	40 annual observations	Economic Environmental	GDP per capita (constant 2010 US$): Gdppc Food production index: Fpi Urbanization (% of total population): Urb CO_2_ emissions (kt): Co2	Adjusted Gdppc β = 0.327965 (*p* = 0.04354) Fpi β = −0.341322 (*p* = 0.08344) Urb β = 0.110738 (*p* = 0.01440) Co2 β = −0.167716 (*p* = 0.04266)
Çavmak et al., 2024 [56]	2024	Longitudinal Time series	Turkey	Upper-Middle-income economies ($4496 to $13,935)	2000–2019 (20)	Turks	20 annual observations	Social Economic Healthcare access/financing Behavioural	Gross domestic product: GDP Enrolment rate in tertiary education: HE Out-of-pocket health expenditure: OOPHE Tobacco consumption (grams per capita): TC	Adjusted GDP β = 0.212 (*p* < 0.01) HE β = 0.129 (*p* < 0.01) OOPHE β = 0.073 (*p* < 0.01) TC β = −0.005 (*p* < 0.01)
Hamidi et al., 2018 [57]	2018	Ecological Time series–Panel data	18 MENA countries (all are OIC members: Algeria, Bahrain, Djibouti, Egypt, Iran, Iraq, Jordan, Kuwait, Lebanon, Morocco, Oman, Qatar, Saudi Arabia, Syria, Tunisia, Turkey, UAE, and Yemen)	Mixed	1995–2009 (15)	Country–year national indicators	270 country-year observations (18 × 15)	Economic Health/disease burden Environmental/infrastructure Health system resources Urbanization Education/human capital	GDP per capita: PPP Tuberculosis incidence Improved water source (% access) Hospital beds (per 1000) Urban population (%) Educational attainment	GDP: Model 6: 0.0166 (*p* < 0.001); Model 7: 0.0159 (*p* < 0.01); Model 8: 0.0241 (*p* < 0.001); Model 9: 0.0190 (*p* < 0.001); Model 10: 0.0223 (*p* < 0.001) TB incidence: Model 6: −0.0110 (*p* ≥ 0.05); Model 7: −0.0096 (*p* ≥ 0.05); Model 8: −0.0099 (*p* ≥ 0.05);; Model 9: −0.0094 (*p* ≥ 0.05); Model 10: −0.0097 (*p* ≥ 0.05) Improved water: Model 6: 0.1060 (*p* < 0.001); Model 7: 0.1198 (*p* < 0.001); Model 8: 0.1227 (*p* < 0.001); Model 9: 0.1252 (*p* < 0.001); Model 10: 0.1203 (*p* < 0.001) Hospital beds: Model 6: 0.0107 (*p* < 0.05); Model 7: 0.0113 (*p* < 0.05); Model 8: 0.0046 (*p* ≥ 0.05); Model 9: 0.0083 (*p* ≥ 0.05); Model 10: 0.0061 (*p* ≥ 0.05) Urban population: Model 6: 0.0360 (*p* ≥ 0.05); Model 7: 0.0891 (*p* < 0.05); Model 8: 0.1160 (*p* < 0.01); Model 9: 0.1216 (*p* < 0.01); Model 10: 0.1200 (*p* < 0.01) Education attainment Men 25–34 (Model 6): 0.1429 (*p* < 0.001); Men ≥ 25 (Model 7): 0.0737 (*p* < 0.001) Women 25–34 (Model 8): 0.0393 (*p* < 0.001); Women ≥ 25 (Model 9): 0.0336 (*p* < 0.001); Women 15–44 (Model 10): 0.0426 (*p* < 0.001)
Saidmamatov et al., 2024 [58]	2024	Panel data econometric study	Aral Sea Basin countries include OIC members (Uzbekistan, Tajikistan, Turkmenistan, Afghanistan, Iran, Kazakhstan, and Kyrgyz Republic)	Mixed	2002–2020 (19)	Country–year national indicators	Panel observations: 133 for most variables; regression uses 108–111 observations (missingness, esp. human capital)	Environment Health system/financing Economic development Water/resources Agriculture/food system Urbanization/settlement Energy Energy transition Education/human capital	CO_2_ emissions (metric tons per capita): CO_2_ Health expenditure (% of GDP): Health Economic growth proxied by GDP: Gdp Water productivity (hectares per person): Water Agricultural value added (% of GDP): Agr Urbanization rate (%): Urb Total energy consumption (kWh): Eng Renewable energy consumption (% of total energy consumed): Re Human capital proxied by primary school enrolment percentage (gross): Hc	Crude CO_2_ r = 0.7341 Health r = 0.8072 Gdp r = 0.5274 Water r = 0.5734 Agr r = −0.5195 Urb r = 0.7513 Energy r = 0.8516 Renew r = −0.4843 Hc r = 0.2319 Adjusted CO_2_: OLS β = −0.0509 (*p* < 0.01), FMOLS β = −0.0465 (*p* < 0.01), DOLS β = −0.0483 (*p* < 0.05), CCR β = −0.047 (*p* < 0.01), Driscoll-Kraay β = −0.0509 (*p* < 0.01) Health: OLS β = 0.0321 (*p* < 0.01), FMOLS β = 0.0534 (*p* < 0.01), DOLS β = 0.0772 (*p* < 0.01), CCR β = 0.0532 (*p* < 0.01), Driscoll–Kraay β = 0.0321 (*p* < 0.01) GDP: OLS β = 0.00188 (*p* ≥ 0.10), FMOLS β = 0.0141 (*p* < 0.10), DOLS β = 0.0332 (*p* ≥ 0.10), CCR β = 0.0138 (*p* ≥ 0.10), Driscoll–Kraay β = 0.00188 (*p* ≥ 0.10) Water: OLS β = 0.0344 (*p* < 0.01), FMOLS β = 0.0250 (*p* < 0.05), DOLS β = 0.00356 (*p* ≥ 0.10), CCR β = 0.0254 (*p* < 0.10), Driscoll–Kraay β = 0.0344 (*p* < 0.05) Agr: OLS β = 0.0208 (*p* < 0.05), FMOLS β = 0.0179 (*p* < 0.05), DOLS β = −0.00468 (*p* ≥ 0.10), CCR β = 0.0177 (*p* < 0.10), Driscoll–Kraay β = 0.0208 (*p* < 0.01) Urb: OLS β = 0.0666 (*p* < 0.10), FMOLS β = −0.0306 (*p* ≥ 0.10), DOLS β = −0.0515 (*p* ≥ 0.10), CCR β = −0.0284 (*p* ≥ 0.10), Driscoll–Kraay β = 0.0666 (*p* < 0.10) Energy: OLS β = 0.0524 (*p* < 0.01), FMOLS β = 0.0576 (*p* < 0.01), DOLS β = 0.0441 (*p* < 0.01), CCR β = 0.0575 (*p* < 0.01), Driscoll-Kraay β = 0.0524 (*p* < 0.01) Renew: OLS β = 0.00298 (*p* ≥ 0.10), FMOLS β = 0.000570 (*p* ≥ 0.10), DOLS β = 0.00273 (*p* ≥ 0.10), CCR β = 0.000355 (*p* ≥ 0.10), Driscoll-Kraay β = 0.00298 (*p* ≥ 0.10) Hc: OLS β = 0.103 (*p* < 0.10), FMOLS β = 0.233 (*p* < 0.01), DOLS β = 0.0716 (*p* ≥ 0.10), CCR β = 0.226 (*p* < 0.01), Driscoll-Kraay β = 0.103 (*p* < 0.10)
Kristanto et al., 2019 [59]	2019	Subnational panel regression	Indonesia	Upper-Middle-income economies ($4496 to $13,935)	2010–2016 (7)	Indonesians	238 province-year observations (34 × 7)	Health infrastructure Socio-economic status	Health personnel Health facilities Health insurance Dependency ratio Income inequality Poverty	Adjusted Health personnel: β = 0.005832 (*p* < 0.01) Health facilities: β = 0.005164 (*p* = 0.3570) Health insurance: β = 0.005259 (*p* < 0.01) Dependency ratio: β = −0.030217 (*p* < 0.01) Income inequality: β = −0.000990 (*p* = 0.8091) Poverty: β = −0.026126 (*p* < 0.01)
Pourshahri et al., 2022 [60]	2022	Population-based cross-sectional study	Iran	Upper-Middle-income economies ($4496 to $13,935)	Feb 2021-Apr 2022 (1.25)	General population residents aged 15–70 years	300 participants	Demographic Education Economic status Household risk context Social Health status Behavioural Occupation COVID severity COVID status	Age Sex (Female vs. Male) Education (Secondary vs. Primary) Education (University vs. Primary) Income (Below Sufficient vs. Sufficient) Income (More than sufficient vs. Sufficient) High-risk at home (Elderly vs. Child) High-risk at home (Underlying-disease person vs. Child) High-risk at home (None vs. Child) Single vs. Married Underlying disease (Yes vs. No) Smoking (Yes vs. No) Employee vs. Other Student vs. Other No hospital admission vs. Yes admission No COVID history vs. COVID history	Unadjusted (Crude) Demographic Age: B = −0.12 (*p* < 0.001) Sex: Female vs. Male: B = −0.59 (*p* = 0.38) Education Secondary vs. Primary: B = −1.86 (*p* = 0.18) University vs. Primary: B = 0.37 (*p* = 0.68) Income Below sufficient vs. Sufficient: B = −3.07 (*p* < 0.001) More than sufficient vs. Sufficient: B = 3.77 (*p* = 0.002) Household risk context High-risk at home: Elderly vs. Child: B = −4.62 (*p* < 0.001) Underlying-disease person vs. Child: B = −2.99 (*p* = 0.003) High-risk at home: None vs. Child: B = −1.52 (*p* = 0.069) Social Single vs. Married: B = 1.24 (*p* = 0.087) Health status Underlying disease: Yes vs. No: B = −4.27 (*p* < 0.001) Behavioral Smoking: Yes vs. No: B = −4.84 (*p* < 0.001) Occupation Employee vs. Other (housewife/unemployed/retired): B = −0.36 (*p* = 0.65) Student vs. Other (housewife/unemployed/retired): B = 1.42 (*p* = 0.088) COVID severity No hospital admission vs. Yes admission: B = 2.7 (*p* = 0.16) COVID status No COVID history vs. COVID history: B = 3.41 (*p* < 0.001) Adjusted Demographic Age: B = −0.26 (*p* = 0.59) Income Below sufficient vs. Sufficient: B = −1.27 (*p* = 0.15) More than sufficient vs. Sufficient: B = 4.86 (*p* < 0.001) High-risk at home Elderly vs. Child: B = −1.69 (*p* = 0.069) Underlying-disease person vs. Child: B = −1.37 (*p* = 0.14) None vs. Child: B = 1.53 (*p* = 0.062) Social Single vs. Married: B = 0.42 (*p* = 0.66) Health status Underlying disease: Yes vs. No: B = −3.47 (*p* < 0.001) Behavioral Smoking: Yes vs. No: B = −2.85 (*p* = 0.022) Occupation Employee vs. Other: B = 0.03 (*p* = 0.96) Student vs. Other: B = −0.87 (*p* = 0.39) COVID status No COVID history vs. COVID history: B = 2.95 (*p* < 0.001)
Esmaeili et al., 2011 [61]	2011	Cross sectional (cross-country)	24 Islamic/OIC countries: Egypt, Gambia, Guyana, Indonesia, Iran, Jordan, Kazakhstan, Kyrgyzstan, Malaysia, Mali, Mauritania, Morocco, Mozambique, Niger, Nigeria, Uzbekistan, Pakistan, Senegal, Tajikistan, Tunisia, Turkmenistan, Turkey, Uganda, Yemen	Mixed	1996–2004 (9)	Country-level national indicators	24 countries	Prosperity Income Education level Environment factors Health care Women’s role	Prosperity: (GDP) Income: (Gini) Environmental factors: Percentage of urban population (Urban) Healthcare/expenditure: (Health) Education: Enrolment ratio in high school (High) Enrolment ratio in university (Univ) Adult literacy rate (Lit) Women’s role: Share of females in the working population (Female)	Adjusted (Model-based) GDP: Equation (4): β = 0.005 (*p* = 0.01); Equation (3): β = −0.8 (ns); Equation (6): β = 0.001 (ns) Gini: Equation (3): β = 0.003 (*p* = 0.01); Equation (1): β = −0.46 (ns); Equation (6): β = −0.32 (ns) High: Equation q(3): β = 0.33 (*p* = 0.01); Equation (6): β = 0.48 (*p* = 0.05) Univ: Equation (6): β = 0.69 (*p* = 0.05) Urban: Equation (6): β = 0.36 (*p* = 0.05) Health: Equation (4): β = −0.27 (ns) Lit: Equation (4): β = 0.11 (ns) Female: Equation (4): β = −0.14 (ns)
Nathaniel & Khan, 2020 [62]	2020	Time series econometric study	Nigeria	Lower-Middle income economies ($1136 to $4495)	1970–2014 (45)	Nigerian	45 annual observations	Environment Urbanization Public finance/health system Economic	CO_2_ emissions (metric tons per capita): CO_2_ Urbanization (% of total population): UBP Public health expenditure: PHE Per-capita income (constant 2010 USD): PCI	Crude CO_2_: r = 0.745 PCI: r = 0.459 UBP: r = 0.926 PHE: r = 0.809 Adjusted CO_2_: β = −0.0378 (ns) PCI: β = 0.0483 (ns) UBP: β = −0.3726 (*p* < 0.01) PHE: β = 0.0155 (*p* < 0.05)
Agbanike et al., 2019 [63]	2019	Ecological study/Time series	Nigeria	Lower-Middle income economies ($1136 to $4495)	1971–2014 (44)	Nigerian	44 annual observations	Environmental Economic Oil sector Financial development Macroeconomic stability Demographic Trade Structural breaks	CO_2_ emissions: CO_2_ GDP per capita: Rgdpc Values of petroleum exports (m $): VPet Daily crude oil production (average): Crdoilp Private credit by deposit money banks to GDP (%): Pcrd Domestic credit to private sector (% of GDP): Dcrd Population Growth (Annual %): Popgrt Inflation, consumer prices (annual %): Infrt Trade (Export + Import % of GDP): Trd	Adjusted: Model 1 (includes VPet, Pcrd, Popgrt) Rgdpc: Total LE β = 0.1663 (*p* < 0.01), LEM β = 0.1151 (ns), LEF β = 0.2528 (*p* < 0.01) CO_2_: Total LE β = −0.0438 (*p* < 0.01), LEM β = −0.0596 (*p* < 0.05), LEF β = −0.0262 (*p* < 0.10) VPet: Total LE β = 0.0714 (*p* < 0.01), LEM β = 0.1076 (*p* < 0.05), LEF β = 0.0457 (*p* < 0.01) Pcrd: Total LE β = 0.0831 (*p* < 0.01), LEM β = 0.0698 (*p* < 0.05), LEF β = 0.0912 (*p* < 0.01) Infrt: Total LE β = −0.0094 (ns), LEM β = −0.0281 (ns), LEF β = 0.0013 (ns) Popgrt: Total LE β = 0.0069 (ns), LEM β = 0.1176 (ns), LEF β = −0.0552 (ns) Model 2 (includes Crdoilp, Dcrd, Trd) Rgdpc: Total LE β = 0.3021 (*p* < 0.01), LEM β = 0.3069 (*p* < 0.01), LEF β = 0.3006 (*p* < 0.01) CO_2_:Total LE β = −0.0674 (*p* < 0.05), LEM β = −0.0784 (*p* < 0.05), LEF β = −0.0346 (*p* < 0.01) Crdoilp: Total LE β = −0.1276 (*p* < 0.10), LEM β = −0.1426 (*p* < 0.10), LEF β = −0.0782 (*p* < 0.01) Dcrd: Total LE β = 0.1020 (*p* < 0.01), LEM β = 0.1151 (*p* < 0.01), LEF β = 0.0905 (*p* < 0.01) lnInfrt: Total LE β = −0.0261 (*p* < 0.10), LEM β = −0.0432 (*p* < 0.05), LEF β = −0.0077 (ns) Trd: Total LE β = −0.0283 (ns), LEM β = −0.0274 (ns), LEF β = −0.0304 (*p* < 0.01)
Aalipour et al., 2023 [64]	2023	Time series econometric study	Iran	Upper-Middle-income economies ($4496 to $13,935)	1981–2020 (40)	Iranians	40 annual observations	Economic Investment/health financing Education Health burden Urbanization Macroeconomic Financial/policy rate	Gross domestic production: GDP foreign direct investment: FDI Literacy rate: LR Health burden: HIV Urbanization: URBEN Real exchange rate: EXR Inflation: INF Interest rate: IR	Adjusted GDP: β = 0.089229 (*p* = 0.067) FDI: β = 0.76302 (*p* = 0.067) LR: β = 1.0230 (*p* = 0.000) HIV: β = −1.7498 (*p* = 0.012) URBEN: β = 2.8264 (*p* = 0.023) INF: β = −0.011868 (*p* = 0.459) IR: β = 0.14316 (*p* = 0.183)
Kanat et al., 2023 [65]	2024	Time series econometric study	Kazakhstan	Upper-Middle-income economies ($4496 to $13,935)	1990–2022 (33)	Kazakh/Kazakhstanis	33 annual observations	Energy use Air pollution/air quality proxy Economic growth Health expenditure Population	Energy use (EU) Air pollution (AP) Economic growth (EG) Health expenditure (HEXP) Population (POP)	Crude EU: r = 0.5300 AP: r = 0.4397 EG: r = 0.4727 HEXP: r = 0.3915 POP: r = 0.5251 Adjusted EU: β = −0.0942 (*p* = 0.0070) AP: β = −0.1294 (*p* = 0.0134) EG: β = 0.04445 (*p* = 0.0213) HEXP: β = 0.01343 (*p* = 0.3932) POP: β = 0.8799 (*p* = 0.0000)
Igbinedion, 2019 [66]	2019	Ecological study/Time series econometric study	Nigeria	Lower-Middle income economies ($1136 to $4495)	1990–2016 (27)	Nigerian	27 annual observations	Environmental Water & sanitation Health financing Mortality/health burden	Carbon dioxide emissions per capita (CDE) Improved sanitation facilities (IMS) Government health expenditure (TGHE) Mortality rate (MRATE)	Crude CDE: r = 0.2081 TGHE: r = 0.2602 IMS: r = −0.3709 MRATE: r = −0.1423 Adjusted CDE: β = −0.968 (*p* = 0.034) IMS: β = 3.279 (*p* = 0.015) HEXP: β = 0.328 (*p* = 0.006) MRATE: β = 52.286 (*p* = 0.000) ECM: β = −0.468 (*p* = 0.034)
Okogor, 2022 [67]	2022	Ecological study/Time series econometric study	Nigeria	Lower-Middle income economies ($1136 to $4495)	1990–2015 (26)	Nigerian	26 annual observations	Environment/pollution Water access Sanitation access Water + sanitation Economic Demographic	CO_2_ emissions per capita (CO_2_) Access to improved water source (AIWS) Access to improved sanitary facility (AISF) Linear combination/average of improved water + sanitation (AIWSISF) GDP per capita (PGDP) Population growth (POP_GRT)	CO_2_: β = −0.009257 (*p* = 0.0117) AIWS: β = −0.845205 (*p* = 0.0001) AISF: β = −0.727756 (*p* = 0.0000) AIWSISF: β = 3.149383 (*p* = 0.0000) PGDP: β = 0.083608 (*p* = 0.0011) POP_GRT: β = 0.108341 (*p* = 0.3409) (ns)
Awan et al., 2024 [68]	2024	Ecological study/Time series	Pakistan	Lower-Middle income economies ($1136 to $4495)	2000 Q1–2020 Q4 (25)	Pakistanis	84 quarters	Environment (land/forests) Climate Environment (air pollution) Demographic/urban context	Deforestation (DEF; tree cover loss) Temperature (TEM) Rainfall (RF) CO_2_ emissions per capita (CO_2_) Urbanization (URB; % urban population)	Crude DEF: r = 0.0729 (*p* ≥ 0.05; ns) TEM: r = 0.0015 (*p* ≥ 0.05; ns) RF: r = 0.2773 (*p* < 0.01) CO_2_: r = 0.3828 (*p* < 0.01) URB: r = 0.3550 (*p* < 0.01) Adjusted DEF: β = 0.0056 (*p* = 0.0347) TEM: β = 0.0042 (*p* = 0.0632) RF: β = 0.0070 (*p* = 0.0354) CO_2_: β = 0.0148 (*p* = 0.0000) URB: β = 0.6846 (*p* = 0.0000)
M. Arafat et al., 2022 [69]	2022	Time series econometric study	Pakistan	Lower-Middle income economies ($1136 to $4495)	1965–2019 (55)	Pakistanis	55 annual observations	Energy Environmental Financial	Energy consumption (EC) Environmental degradation (ED) Financial development (FD)	EC: β = 2.05 (*p* < 0.01); DOLS β = 0.32 (*p* < 0.01) ED: β = −1.77 (*p* < 0.01); DOLS β = −0.23 (*p* < 0.01) FD: β = 0.65 (*p* < 0.01); DOLS β = 0.05 (*p* < 0.01)
Abbas et al., 2024 [70]	2024	Ecological study/Time series	Pakistan	Lower-Middle income economies ($1136 to $4495)	1965–2020 (56)	Pakistanis	56 annual observations	Environmental (air pollution) Economic Health financing/fiscal	CO_2_ emissions (CO_2_) GDP per capita (GDPpc) Current health expenditure per capita (CHE)	Crude CO_2_: r = 0.9293 (*p* < 0.01) GDPpc: r = 0.8790 (*p* < 0.01) CHE: r = 0.9504 (*p* < 0.01) Adjusted CO_2_: β = −2.150 (*p* ≥ 0.1) GDPpc: β = 7.730 (*p* < 0.01) CHE: β = 0.040 (*p* ≥ 0.1)
Omri et al., 2022 [71]	2022	Ecological study/Time series	Saudi Arabia	High-income economies ($13,935 or more)	2000–2018 (19)	Saudi Arabia	19 annual observations	Health financing Research/innovation Environment Socioeconomic Energy & trade	Government health expenditure (HE, % GDP) R&D expenditure (RDexp, % GDP) Environmental-related patents (PET) CO_2_ indicators (COpc) (per capita) Electricity/heat (COehp) Liquid fuel (COlfc) CO_2_ intensity (COint) GDP per capita (Y) Tertiary enrolment (Edu) Energy use (EC) Trade openness (Tr)	HE: β = 0.144 (*p* = 0.011) RDexp: β = 0.074 (*p* = 0.023) Edu: β = 0.303 (*p* = 0.000) Y (GDPpc): β = 0.197 (*p* = 0.000) COpc: β = 0.123 (*p* = 0.118) (ns) COehp: β = 0.102 (*p* = 0.156) (ns) COlfc: β = 0.072 (*p* = 0.209) (ns) COint: β = 0.105 (*p* = 0.1325) (ns)
Hussein et al., 2024 [72]	2024	Ecological study/Time series	Somalia	Low-income economies ($1135 or less)	1990–2020 (31)	Somalis	31 annual observations	Environmental/pollution Trade/openness Demographic Capital/investment Economic growth (outcome)	CO_2_ emissions, kilotons (CO_2_) Trade openness: (TO) Population growth (PG) Gross capital formation (CAPITAL) Real GDP per capita (RGDPC)	LE: β = 0.809 (*p* = 0.031)
Nandi et al., 2023 [73]	2023	Ecological study/Time series	Bangladesh	Lower-Middle income economies ($1136 to $4495)	1991–2019 (29)	Bangladeshis	29 annual observations	Economic Labor market Demographic	Gross National Income (GNI, current US$) Unemployment rate (% of total labor force) Employment rate (% of total employment) Population growth rate (annual %) Age dependency ratio (% of working-age population)	Crude GNI: r = 0.436 (*p* < 0.01) Unemployment: r = −0.411 (*p* < 0.05) Employment: r = 0.558 (*p* < 0.01) Population growth: r = −0.443 (*p* < 0.01) Age dependency: r = −0.393 (*p* < 0.05) Adjusted GNI: β = 0.436 (*p* < 0.01) Unemployment: β = −0.411 (*p* < 0.05) Employment: β = 0.558 (*p* < 0.01) Population growth: β = −0.443 (*p* < 0.01) Age dependency: β = −0.393 (*p* < 0.05)
Setiawan et al., 2023 [74]	2023	Ecological study/Time series	Indonesia	Upper-Middle-income economies ($4496 to $13,935)	1990–2021 (32)	Indonesians	32 annual observations	Economic Health financing Environment/emissions Mortality/health burden Socioeconomic	Economic growth (EG = GDP per capita) Health expenditure (Hex) Carbon emission per capita (Emc) Mortality rate (Mor) Poverty rate (Pov)	Adjusted EG: β = 0.002944 (*p* < 0.05) Hex: β = 3.982365 (*p* < 0.01) Emc: β = −2.673902 (*p* > 0.05; ns) Mor: β = −1.767353 (*p* > 0.05; ns) Pov: β = −6.820181 (*p* < 0.05)
Ghaedrahmati & Hajilou, 2022 [75]	2022	Ecological study/Time series	Iran	Upper-Middle-income economies ($4496 to $13,935)	2000–2020 (21)	Iranians (Tehran city)	21 annual observations	Air pollution	PM10, PM2.5, CO, O_3_, SO_2_, NO_2_	Crude CO: r = −0.944, *p* = 0.000 O_3_: r = 0.504, *p* = 0.012 NO_2_: r = 0.945, *p* = 0.000 SO_2_: r = −0.821, *p* = 0.000 PM10: r = −0.255, *p* = 0.132 PM2.5: r = −0.879, *p* = 0.000 Adjusted CO: B = −0.022; β = −0.140; *p* = 0.000; 95% CI [−0.098, 0.053] O_3_: B = 0.046; β = 0.218; *p* = 0.000; 95% CI [−0.030, 0.122] NO_2_: B = 0.036; β = 0.248; *p* = 0.000; 95% CI [−0.083, 0.155] SO_2_: B = −0.094; β = −0.803; *p* = 0.000; 95% CI [−0.440, 0.252] PM10: B = −0.225; β = −0.773; *p* = 0.000; 95% CI [−0.734, 0.285] PM2.5: B = 0.107; β = 0.861; *p* = 0.000; 95% CI [−0.361, 0.574]
Adeshina et al., 2019 [76]	2019	Ecological study/Time series	Nigeria	Lower-Middle income economies ($1136 to $4495)	1981–2017 (37)	Nigerian	37 annual observations	Fiscal policy/public spending Monetary policy/financial sector Fiscal policy/debt Macroeconomic stability	Total public capital expenditure (LNTPCE) Financial deepening (FD = MS/GDP) Domestic debt (LNDD) Inflation rate (INF)	OLS LNTPCE: β = −0.017968 (*p* = 0.0135) FD: β = 0.007637 (*p* = 0.0000) LNDD: β = 0.029406 (*p* = 0.0005) INF: β = −0.000589 (*p* = 0.0031) ARDL LNTPCE: β = −0.066068 (*p* = 0.0008) FD: β = 0.012776 (*p* = 0.0002) LNDD: β = 0.062764 (*p* = 0.0004) INF: β = 0.002022 (*p* = 0.0860) (ns)
Wirayuda, Jaju et al., 2022 [77]	2022	Ecological study/Time series	Oman	High-income economies ($13,935 or more)	1978–2018 (41)	Omanis	41 annual observations	Sociodemographic (SD) Macroeconomic (ME) Health status & resources (HSR)	Infant mortality rate (IMR) Fertility rate (FR) Adult mortality—female (AM(f)) GDP per capita (GDP) Dependency ratio (DR) Capital investment (CI) CO_2_ emissions (CO_2_E) Mental & substance use disorders (MSU) Obesity prevalence—female (O(f)) Obesity prevalence—male (O(m))	Sociodemographic (SD) Primary school enrolment (PSE) PSE: r = 0.99 (*p* < 0.01) Crude SSE: r = 0.99 (*p* < 0.01) IMR: r = 0.99 (*p* < 0.01) FR: r = −0.97 (*p* < 0.01) AM(f): r = −0.94 (*p* < 0.01) GDP: r = 0.79 (*p* < 0.01) DR: r = −0.95 (*p* < 0.01) CI: r = −0.76 (*p* < 0.01) CO_2_E: r = 0.62 (*p* < 0.01) MSU: r = 0.42 (*p* < 0.01) O(f): r = 0.39 (*p* < 0.05) O(m): r = 0.72 (*p* < 0.01) Adjusted SD → LE: β = −0.92 (*p* < 0.001) ME → LE: β = −0.15 (*p* < 0.001) HSR → LE: β = 0.23 (*p* < 0.001)

β = regression coefficient; *r* = correlation coefficient. Significant values are marked with an asterisk (*) at the 5% level.

### 4.2. Macroeconomic and Economic Determinants

Table 2 shows that higher income and economic development were generally associated with longer LE. In Nigeria, time-series analyses consistently showed positive effects of income. For example, in one macroeconomic model, inflation (β = −0.034493, *p* < 0.001) and imports (β = −0.068840, *p* < 0.001) were negatively related to LE [26]. Likewise, LE was positively correlated with per capita GDP (β = 0.140123, *p* < 0.001), whereas poverty indicators like poverty headcount ratio (indicates the percentage of the population living below the poverty line [78] (β = −0.1672, *p* < 0.001), poverty gap (β = −0.1401, *p* = 0.0011), and squared poverty gap (β = −0.1223, *p* = 0.0026) were inversely related to LE [27]. In another Nigerian study, real GDP per capita (β = 0.4132; *p* = 0.0150) showed a significant positive association with LE [29].

The significance of income was also highlighted in a multi-country analysis for the OIC or regional groupings. For 46 OIC member nations between 2010 and 2018, log GDP per capita (LN_GDP) was positively and highly correlated with LE (β = 6.019235, *p* < 0.001), alongside favorable effects of health expenditure and schooling [30]. In the Eastern Mediterranean Region (21 countries), GDP per capita (β = 0.0229, *p* = 0.011) was positively associated with LE [31]. Structural equation modeling for Indonesia and Oman reported sizeable total effects of the macroeconomic (ME) construct on LE; for example, ME → LE (β = 0.848, 95% CI 0.784–0.899) for Oman and (β = 0.737, 95% CI 0.527–0.904) for Indonesia [34], with similar positive paths in GCC-wide models [35].

In Pakistan, per capita income generally showed a positive association with LE. One ARDL study found that per capita income (β = 0.001144, *p* = 0.8812) had a small, non-significant coefficient in the baseline model. However, it was strongly positive in robustness checks using FMOLS (β = 0.024526, *p* < 0.001) and DOLS (β = 0.019516, *p* = 0.0532) [36]. Another Pakistani time-series analysis reported a positive association between GDP per capita (β = 7.730, *p* < 0.01) and LE in the adjusted model [70]. In Nigeria, poverty measures were robustly negatively related to LE, with the poverty headcount (β = −0.1672, *p* < 0.001), poverty gap (β = −0.1401, *p* = 0.0011), and squared poverty gap (β = −0.1223, *p* = 0.0026) all showing significant associations [27]. Income inequality (GINI) in Pakistan was also negatively associated with LE (β = −0.25060, *p* = 0.0044), while GDP per capita (β = 0.02238, *p* < 0.001) remained strongly positive [37]. In Nigeria, income inequality was negatively associated with LE (β = −0.1946, *p* = 0.0293) [47].

Other country-specific studies showed similar patterns. In Bangladesh, gross national income and labor-market indicators were significantly associated with LE in both crude and adjusted models: GNI (β = 0.436, *p* < 0.01) [73]. In Lebanon, GDP per capita (β = 0.001062, *p* < 0.001) was positively associated with LE [48]. For MENA OIC countries, GDP per capita (β ≈ 0.0166–0.0241, *p* ≤ 0.01) retained a consistently positive and statistically significant association with LE across multiple models [57]. In Iran, GDP (β = 0.089229, *p* = 0.067) and foreign direct investment (β = 0.76302, *p* = 0.067) had positive but borderline-significant coefficients [64]. Government capital expenditure, domestic debt, and financial deepening in Nigeria were positively related to LE, for example, ARDL estimates of financial deepening (β = 0.012776, *p* < 0.001) and domestic debt (β = 0.062764, *p* = 0.0004), whereas total public capital expenditure (β = −0.066068, *p* = 0.0008) was negatively associated with LE [76].

### 4.3. Social and Sociodemographic Determinants

Social and sociodemographic factors, especially education, employment, and poverty, showed consistent associations with LE. In the 46-country OIC panel, mean years of schooling had a strong positive effect (β = 0.575393, *p* < 0.001), while smoking prevalence showed a significant negative association (β = −0.220921, *p* < 0.001); unemployment was non-significant (β = −0.009166, *p* = 0.8055) [30].

Education was a key determinant in several studies, for 19 Arab OIC countries, female and male literacy and enrolment rates correlated positively with LE, for example, female enrolment (Enf) r = 0.87 and male enrolment (Enm) r = 0.81 with female LE, and similar magnitudes for male LE [50]. In Malaysia, literacy (r = 0.69, *p* < 0.05) and doctors per capita (r = 0.75, *p* < 0.05) correlated positively with LE, and in a path model, health resources (β = 0.47, *p* < 0.05) had a direct positive effect on LE, with socioeconomic status and demographic factors acting through health resources [51]. In Turkey, secondary-school enrolment was positively associated with LE in both crude (r = 0.9897, *p* < 0.001) and adjusted (β = 0.176652, *p* < 0.001) models [54], and tertiary enrolment in another Turkish study had a positive coefficient (β = 0.129, *p* < 0.01) [56]. In Oman and Indonesia, structural equation models showed that sociodemographic constructs (e.g., enrolment and demographic structure) had sizeable indirect effects on LE via health resources, with SD → LE total effects of β = 0.675–0.755 (all *p* < 0.001) in some models [34].

Labor-market variables also played a role. In Bangladesh, a higher employment rate was positively associated with LE (β = 0.558, *p* < 0.01). In contrast, unemployment (β = −0.411, *p* < 0.05), population growth (β = −0.443, *p* < 0.01), and higher age dependency (β = −0.393, *p* < 0.05) were negative predictors [73].

### 4.4. Environmental Determinants

Environmental factors, particularly air pollution and CO_2_ emissions, were frequently associated with LE in OIC settings. In Nigeria, one study found that CO_2_ emissions (β = −45.0359, *p* = 0.0066) and income inequality (β = −0.1946, *p* = 0.0293) negatively affected LE [47]. Another Nigerian time-series analysis found that CO_2_ was not significant [28]. In Palestine, CO_2_ emissions (β = −0.003, *p* < 0.01) showed a modest negative association with LE. In Lebanon, CO_2_ emissions had a strong negative coefficient (β = −1.072773, *p* = 0.0024) with LE [48].

In Pakistan, several studies examined environmental pressures. Using ARDL, one study found that CO_2_ emissions (β = −0.046395, *p* = 0.0007) were negatively associated with LE in the main model, with similar negative effects in FMOLS (β = −0.007595, *p* = 0.0530), and DOLS (β = −0.013032, *p* = 0.0289) [36]. Another Pakistani analysis focusing on deforestation and climate reported positive crude correlations between LE and rainfall (r = 0.2773, *p* < 0.01), CO_2_ (r = 0.3828, *p* < 0.01) and urbanization (r = 0.3550, *p* < 0.01) and in the adjusted model, deforestation (β = 0.0056, *p* = 0.0347), rainfall (β = 0.0070, *p* = 0.0354), CO_2_ (β = 0.0148, *p* <0.001) and urbanization (β = 0.6846, *p* <0.001) were all positively associated with LE [68]. Another time-series study reported very strong positive crude correlations between LE and CO_2_ (r = 0.9293, *p* < 0.01), but in the adjusted model, it was not significant [70].

Environmental and energy indicators were also important in Gulf and Central Asian settings. A study in GCC countries reported that an ecological footprint deficit (β = −2.5654, *p* = 0.034) was negatively associated with LE in pooled models, while the technological achievement index (β = 88.9262, *p* = 0.015) had a large positive effect [39]. In Saudi Arabia, ecological footprint and CO_2_ emissions were positively associated with LE in adjusted pre- and post-Vision 2030 models (Carbon: β ≈ 0.50–0.63, *p* < 0.005) [40]. In Kazakhstan, energy use (β = −0.0942, *p* = 0.007) and air-pollution proxies (β = −0.1294, *p* = 0.0134) were negatively associated with LE in the adjusted model [65].

Ambient air pollutants were also examined in detail in Iran, where particulate matter and gaseous pollutants showed strong crude and adjusted associations with LE. Crude correlations indicated significant relationships between LE and carbon monoxide (r = −0.944, *p* < 0.001), ozone (r = 0.504, *p* = 0.012), nitrogen dioxide (r = 0.945, *p* < 0.001), SO_2_ (r = −0.821, *p* < 0.001), and PM_2_._5_ (r = −0.879, *p* < 0.001) [75]. In adjusted models, standardized β-coefficients remained sizable, for SO_2_ (β = −0.803, *p* < 0.001) and PM_2_._5_ (β = 0.861, *p* < 0.001).

### 4.5. Health-System Resources, Health Burden, and Related Factors

Health-system resources, expenditure, and disease burden were central in many analyses. In the 46-country OIC panel, health expenditure as a share of GDP showed a positive association with LE (β = 0.132586, *p* = 0.0400) [30]. In Pakistan, health expenditure (β = 0.000215, *p* = 0.0295) was positively associated with LE in ARDL models, although in DOLS the coefficient (β = 0.000372, *p* = 0.4076) became non-significant [36].

In GCC and high-income OIC settings, several structural equation and path analysis papers highlighted the importance of health resources. In GCC countries, crude correlations between LE and health-resource (HR) construct were very strong (pooled HR-LE: r = 0.8940, *p* < 0.05), and in SEM models HR had significant direct effects on LE (HR → LE: β = 0.468, *p* < 0.001, *p* < 0.001) [35]. In Oman and Qatar, health status and resources directly predicted LE, with large coefficients (HSR → LE: β = 0.839 in Oman and β = 0.904 in Qatar). At the same time, sociodemographic and macroeconomic variables influenced LE indirectly through HSR [32]. Similar patterns were observed in models for Indonesia and Oman that decomposed total effects via health resources [34].

Health-system capacity and access also appeared in other settings. In the Eastern Mediterranean Region, crude correlations showed that physician density, vaccination coverage, literacy, safe-water access and urbanization were positively related to LE; in adjusted models, vaccination (β = 0.0018, *p* = 0.026) and urbanization (β = 0.0021, *p* = 0.026) remained significant, whereas health expenditure and physician density did not [31]. In Malaysia, doctors per 10,000 population and government health expenditure had strong positive correlations with LE (Doctor: r = 0.75; Expenditure: r = 0.81; both *p* < 0.05), and health resources (β = 0.47, *p* < 0.05) had a direct positive effect on LE in the path model [51]. In Saudi Arabia, government health expenditure (β = 0.144, *p* = 0.011) was positively associated with LE, alongside tertiary enrolment and GDP per capita [71]. In Palestine, health expenditure per capita (β = 0.002, *p* < 0.01) was positively associated with LE [38].

Studies also highlighted the role of health burden indicators. For 19 Arab OIC countries, infant mortality rate (IMR) and maternal mortality ratio (MMR) were strongly and negatively correlated with both female and male LE (IMR: r = −0.95, MMR: r = −0.94 with female LE; similar magnitudes for male LE), while skilled birth attendance, prenatal care, nutrition, physicians per capita and literacy were positively correlated [50]. In Pakistan, higher death rates and infant mortality were strongly negatively associated with LE: death rate (β = −0.911756, *p* = 0.001), infant mortality (β = 0.178382, *p* = 0.0091) in one specification, where higher infant mortality is coded in an inverse way [36]. In Nigeria, improved sanitation (β = 3.279, *p* = 0.015) and health expenditure (β = 0.328, *p* = 0.006) were positively associated with LE [66]. Disease burden and morbidity indicators, such as tuberculosis incidence, HIV burden, and non-communicable disease measures, also appeared with the expected negative signs in several multi-country and national models, HIV (β = −1.7498, *p* = 0.012) in Iran [57,64].

## 5. Discussion

This review brings together the available evidence on factors associated with LE in OIC countries. Overall, the studies suggest that longevity in these settings is influenced by a combination of economic conditions, social and demographic factors, environmental exposures, and the availability of health-system resources. Several determinants appear consistently across the literature. In particular, higher levels of economic development, better education, and greater health-system capacity are generally associated with longer LE, whereas poverty, inequality, and environmental pollution are often linked to poorer outcomes. The following sections discuss these determinants in more detail.

### 5.1. Economic Development and Macro-Financial Conditions

In this review, economic development emerged as one of the most consistently reported factors associated with LE. In the 46-country OIC panel, higher GDP per capita (log-transformed) showed a large positive adjusted effect on LE, together with health expenditure and schooling [30]. Similar positive adjusted coefficients for GDP or income per capita were observed in time-series models for Pakistan, Bangladesh, Nigeria, and several other OIC settings, even when models also included social, environmental, and fiscal variables [27,73,79]. These findings suggest that higher national income may allow greater investment in nutrition, housing, education, and health services, which are commonly linked with improvements in LE.

The pattern in the OIC region is very consistent with recent cross-country studies in other parts of the world. A longitudinal analysis of 36 OECD countries from 1999 to 2018 found that GDP per capita was one of the most important macro-level determinants of LE at birth, even after controlling for health spending, education, and lifestyle factors [4]. A broader study using 68 determinants in 61 countries over 30 years also identified GDP per capita and demographic structure as key drivers of LE and disability-adjusted life-years [16]. Recent global work on LE confirms that economic prosperity remains a core ingredient of population longevity. However, it is insufficient on its own without progress in social and health systems [80].

At the same time, some results show that not all macroeconomic variables are beneficial. In several Nigerian and Pakistani models, high inflation, income inequality, and various forms of macro-instability were associated with lower LE after adjustment. For instance, one Nigerian study reported negative adjusted coefficients for inflation and income inequality, alongside positive effects of per capita GDP and health expenditure [26,47]. In Pakistan, inequality in the GINI index has a significant negative effect on LE after controlling for GDP and health spending [37]. This result suggests that macroeconomic growth accompanied by high inequality, volatile prices, or weak social protection does not fully translate into health gains. Recent international evidence from high- and middle-income countries shows similar patterns, in which income inequality and insufficient social spending weaken the positive effect of GDP growth on population health and LE [4,80].

Taken together, the evidence suggests that economic development is closely associated with LE in OIC countries. However, the benefits of growth may be reduced in contexts characterized by high inequality or macroeconomic instability.

### 5.2. Social Determinants

This review also found strong evidence that education and broader social conditions are closely linked with LE, even after accounting for income. In the OIC-wide panel, mean years of schooling had a large positive adjusted coefficient, while smoking prevalence showed a strong negative association with LE [30]. In Bangladesh, higher employment and lower unemployment, lower population growth, and lower dependency ratios were significantly associated with longer LE in adjusted models [73]. Poverty intensity in Nigeria remained a significant negative determinant of LE even after controlling for GDP per capita and other macroeconomic factors [27].

These findings are consistent with the wider literature on social determinants of health. A recent global analysis during and after the COVID-19 period showed that combinations of high educational attainment, economic prosperity, social stability, and strong public health capacity were the key configurations associated with high LE across countries [81]. Another recent empirical study focusing on social determinants of health reported that education- and income-related variables remained significant predictors of LE after adjustment for behavioral and healthcare factors [82]. The WHO also continues to highlight that gains in LE are unequal and largely driven by gradients in education, income, working and living conditions, and discrimination [10].

Overall, the findings suggest that social conditions, particularly education, employment opportunities, and poverty levels, are strongly linked with differences in LE across OIC countries.

### 5.3. Environmental Degradation and Air Pollution

A third important finding from this review is the consistent negative effect of environmental degradation, especially air pollution and CO_2_ emissions, on LE in many OIC settings, once other factors are considered. For example, in a Nigerian study, CO_2_ emissions had a large negative adjusted coefficient [47]. In Pakistan, several ARDL and panel models showed that higher CO_2_ emissions were associated with lower LE after adjustment, although the magnitude and significance sometimes varied across estimation [36,69]. In Kazakhstan, energy use and air pollution indicators were negatively associated with LE in adjusted models [65]. Strong negative adjusted associations between particulate air pollution (PM_2_._5_ and PM_10_), Sulphur dioxide, and LE were also reported in an Iranian time-series study [75].

These findings are consistent with recent global evidence on the health impacts of air pollution. A worldwide analysis estimated that ambient air pollution reduces LE by about 2.9 years on average, comparable to or exceeding the impact of tobacco smoking [13]. The State of Global Air report similarly concluded that current levels of fine particulate matter (PM_2_._5_) and household air pollution together reduce global LE by almost two years [12]. More recent panel analyses also show a robust negative association between CO_2_ emissions, other proxies of environmental degradation, and LE across global and regional samples, even after adjusting for GDP and health spending [39,83].

The evidence also indicates that environmental degradation, especially air pollution, is an important factor associated with lower LE in many OIC settings.

### 5.4. Health-System Resources, Expenditure, and Disease Burden

Finally, this review highlights the central role of health-system resources and disease burden in shaping LE. In the OIC panel, health expenditure as a share of GDP had a positive adjusted association with LE, even after controlling for income, education, and inequality [30]. In Pakistan, several studies reported that current health expenditure and health financing variables were positively associated with LE in at least some specifications, although the magnitude and significance varied [79]. In multi-country and GCC-focused structural equation models, health status and resources (for example, physician density, hospital beds, vaccination coverage, and primary care utilization) had strong direct effects on LE, while macroeconomic and sociodemographic factors operated partly through these health-system pathways [35].

Similar associations have also been reported in recent international studies. A comparative study of health and social spending in high-income countries found that higher public health and social expenditure were associated with longer LE and lower mortality, even after adjustment for GDP and demographic structure [84]. Another cross-national analysis showed that healthcare resources, together with economic and demographic factors, are among the most important predictors of LE and DALYs over three decades [16]. At the same time, the WHO and OECD emphasize that there are diminishing returns to very high levels of health spending, and that how money is spent (for example, on primary care, prevention, and financial protection) matters as much as how much is spent [10,85].

All the above findings further highlight the importance of health-system capacity in supporting improvements in population health and LE.

## 6. Strengths and Limitations

A key strength of this review is the use of a pre-registered PROSPERO protocol, PRISMA-guided reporting, JBI appraisal check lists, and a relatively strict quality threshold (≥80%), which increased confidence that the synthesis reflects more robust evidence; in addition, the focus on OIC member countries provides a targeted contribution to a literature often dominated by OECD settings, and the extraction emphasized adjusted estimates to reduce confounding. However, several limitations should be noted. First, most included studies were ecological or time-series analyses using national aggregates, which limits causal interpretation and restricts the findings to associations at the population level. Second, substantial heterogeneity in determinants, data sources, time periods, and modeling approaches reduced comparability and limited quantitative pooling. Third, this review does not include evidence from all OIC member states, and some regions were underrepresented in the available studies. This limitation likely reflects variations in data availability and research output across countries, which may introduce potential bias and limit the generalizability of the findings across all 57 OIC countries. The included studies spanned more than seven decades (1950–2022), during which several countries may have transitioned across low-, middle-, and high-income categories. Consequently, income group designation may not have remained consistent throughout the review period, and the use of a single classification framework may not fully capture the socioeconomic conditions present when the primary studies were undertaken. This temporal variation may have affected the comparability of studies and introduced potential misclassification in the income-based interpretation of the findings. Finally, some articles were not accessible, and the restriction to English-only publication language may have led to the omission of relevant regional evidence.

## 7. Conclusions

Overall, this systematic review shows that LE in OIC countries is shaped by interacting economic, social, environmental, and health-system factors. Higher GDP per capita, better education, stronger employment, and greater health expenditure are consistently associated with longer LE. In contrast, poverty, inequality, air pollution, and limited health resources tend to shorten lives or slow progress. These findings suggest that improvements in LE in OIC countries are likely to be associated with broader progress across economic, social, environmental, and health-system domains, highlighting the importance of coordinated multisectoral policies. Future research should prioritize stronger causal designs and improved country- and subnational-level data to clarify mechanisms and support more targeted interventions.

## Data Availability

All existing data are contained in the manuscript.

## Supplementary Materials

The following supporting information can be downloaded at: https://www.mdpi.com/article/10.3390/ijerph23040531/s1, File S1: Detailed Search Strategy Used in the Databases; File S2: PRISMA checklist.
