# Supplementary material for: The Influence of Social Determinants of Health, Environmental, and Healthcare Resources on Life Expectancy in the Organization of Islamic Cooperation (OIC) Countries: A Systematic Review
Source: Int J Environ Res Public Health. 2026 Apr 18;23(4):531. doi: 10.3390/ijerph23040531 (PMC13115672; doi:10.3390/ijerph23040531)
Supplement: Supplementary file 1 [file ijerph-23-00531-s001.zip › ijerph-4207084-File S1.pdf]

Supplementary File S1

Detailed Search Strategy Used in the Databases

The following keyword-based Boolean search strategies were used to identify studies examining determinants of life expectancy in Organisation of Islamic Cooperation (OIC) member states. Searches combined the outcome (life expectancy) with socioeconomic, macroeconomic, environmental, and health system determinants, as well as country-specific terms. The strategies were adapted to each database's syntax.

A core search structure was developed and applied separately for each of the 57 OIC member states by replacing the country name in the search string. The example below illustrates a MEDLINE search structure.

Search No.	Search Strategy
1	kw:(Life expectancy) AND kw:(socioeconomic status OR socio-demographics OR macroeconomics OR health resources OR environmental OR pollution) AND kw: (Saudi Arabia) [Template used and repeated by replacing "Saudi Arabia" with each OIC country name]
2	kw:(Life expectancy) AND kw:(socioeconomic status OR socio-demographics OR macroeconomics OR health resources OR environmental OR pollution) AND kw:(Oman OR Qatar OR Bahrain OR Saudi Arabia OR Kuwait OR United Arab Emirates)
3	kw:(Life expectancy) AND kw:(Social determinants of health OR Socioeconomics OR macroeconomics OR sociodemographics) AND kw:(Health Resources) AND kw:(Environment) AND kw:(OIC members OR Islamic countries)
4	kw:(Life expectancy) AND kw:(Social determinants of health OR Socioeconomics OR macroeconomics OR sociodemographics OR Health Resources OR Environment) AND kw:(OIC members OR Islamic countries)

Searches were conducted up to 3rd September 2025; The template search using 'Saudi Arabia' was repeated by replacing the country name with each of the 57 OIC member states; Boolean operators (AND, OR) were used to combine outcome, determinant, and country terms; Search syntax was adapted for each database where necessary.
